# Supplementary material for: RaFAH: Host prediction for viruses of Bacteria and Archaea based on protein content
Source: Patterns (N Y). 2021 Jun 15;2(7):100274. doi: 10.1016/j.patter.2021.100274 (PMC8276007; doi:10.1016/j.patter.2021.100274)
Supplement: Document S1. Figures S1–S9 and Data S1–S4 [file mmc1.pdf]

Patterns, Volume *2*

Supplemental information

RaFAH: Host prediction for viruses of Bacteria and Archaea based on protein content

Felipe Hernandes Coutinho, Asier Zaragoza-Solas, Mario López-Pérez, Jakub Barylski, Andrzej Zielezinski, Bas E. Dutilh, Robert Edwards, and Francisco Rodriguez-Valera

Supplementary material:

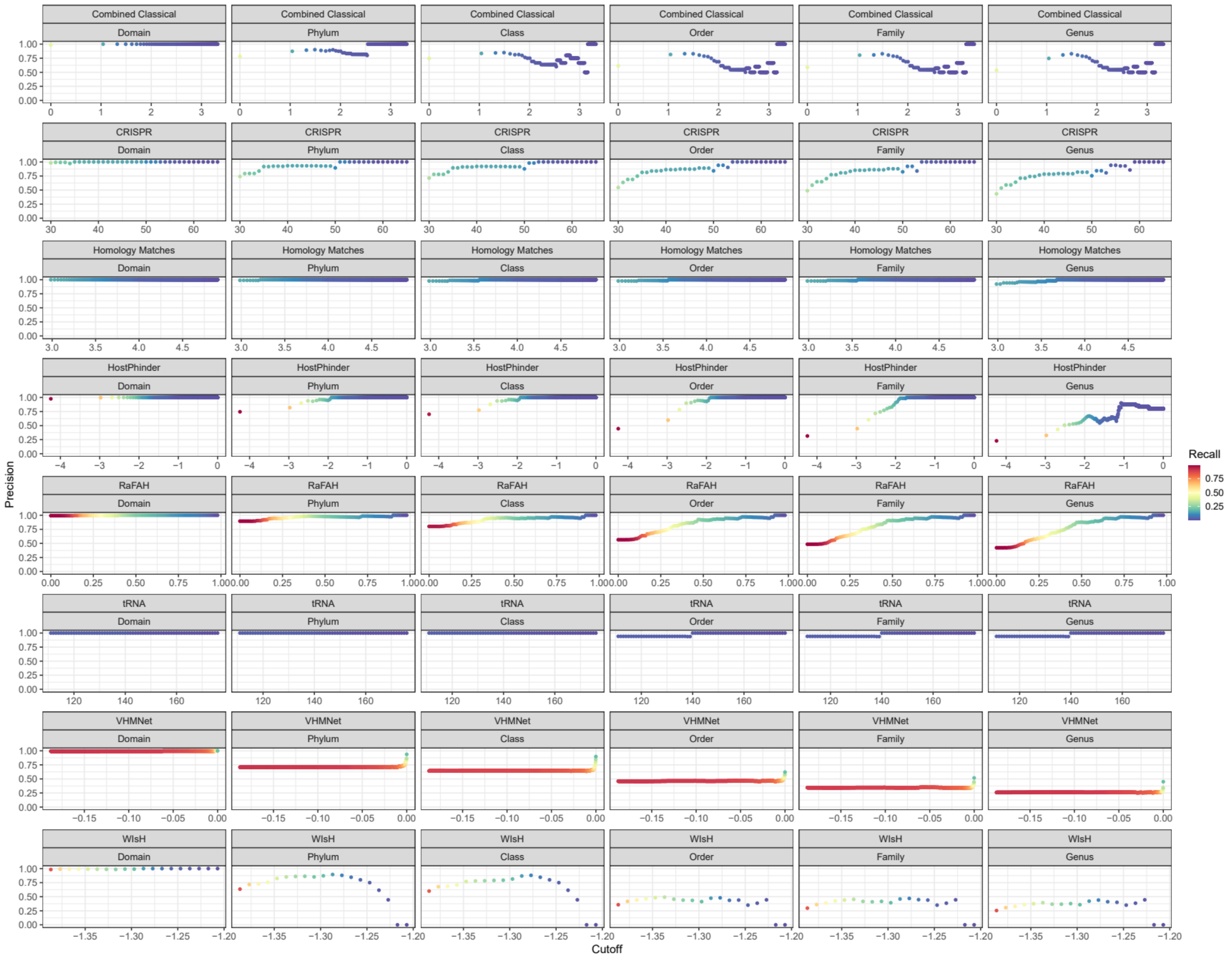

Figure S1: Associations between score cutoff, precision, and recall for RaFAH, the alignment-free (WIsH and HostPhinder), hybrid (VirHostMatcher-Net), and classical (CRISPR, tRNA and homology matches) host prediction approaches on Test Set 1. The score cutoffs for HostPhinder, Homology matches, VirHostMatcher-Net and Combined Classical are shown in the Log10 scale.

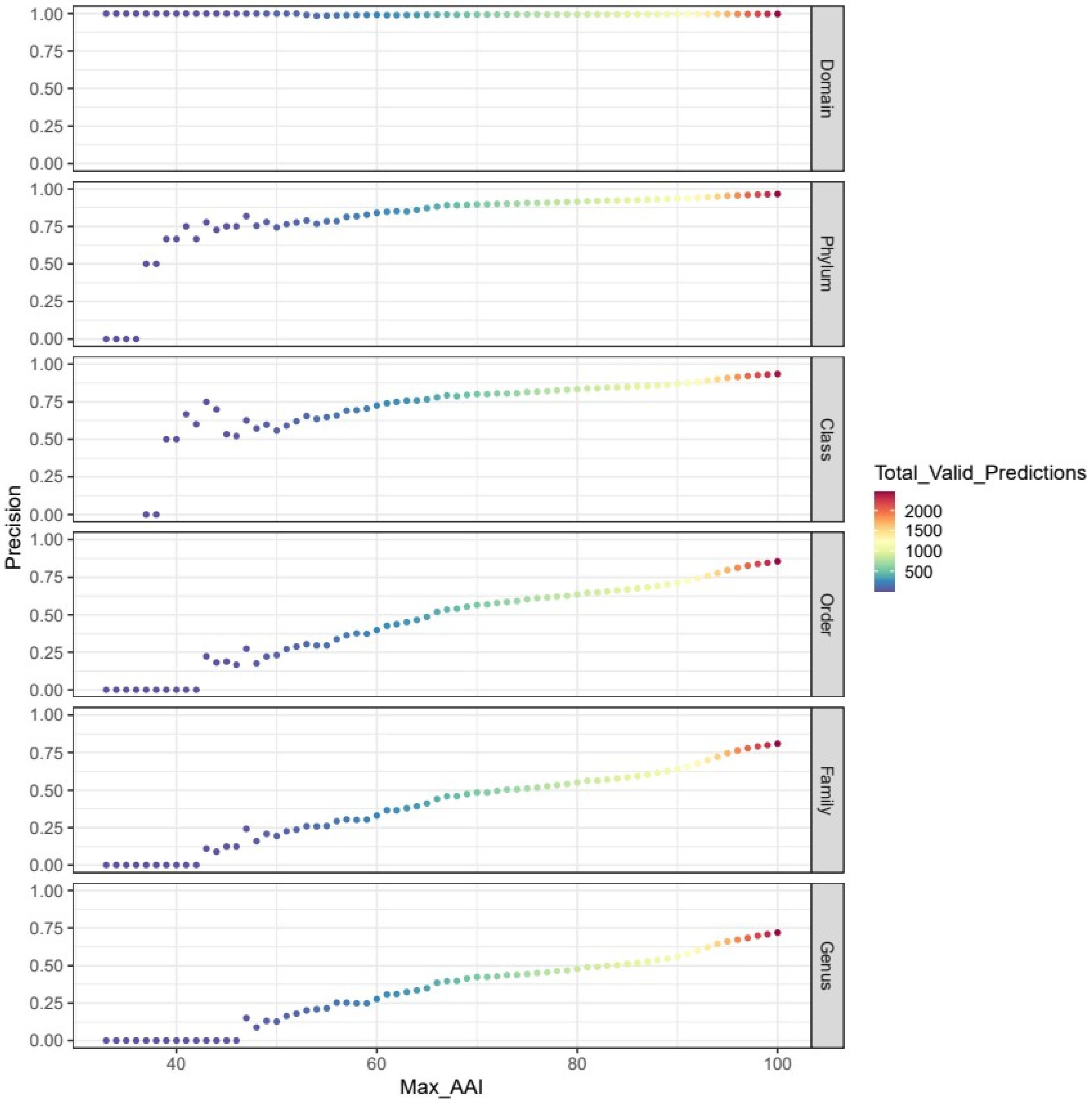

Figure S2: Associations between precision and similarity among Test Set 1 and Training Set 3 genomes. Each panel represents a different taxonomic level. X axis displays the maximum Average Amino acid Identity (AAI) among genomes of the two sets. Y axis displays the precision of RaFAH. Points are coloured according to the number of valid predictions (host taxon predicted by RaFAH for a non "NA/undef/Unknown" host genome in Test Set 1) yielded at each taxonomic level and AAI cutoff. For this particular analysis all non-redundant genomes in Test Set 1 were used while in all other instances this dataset was filtered for maximum 70% AAI and 70% matched proteins.

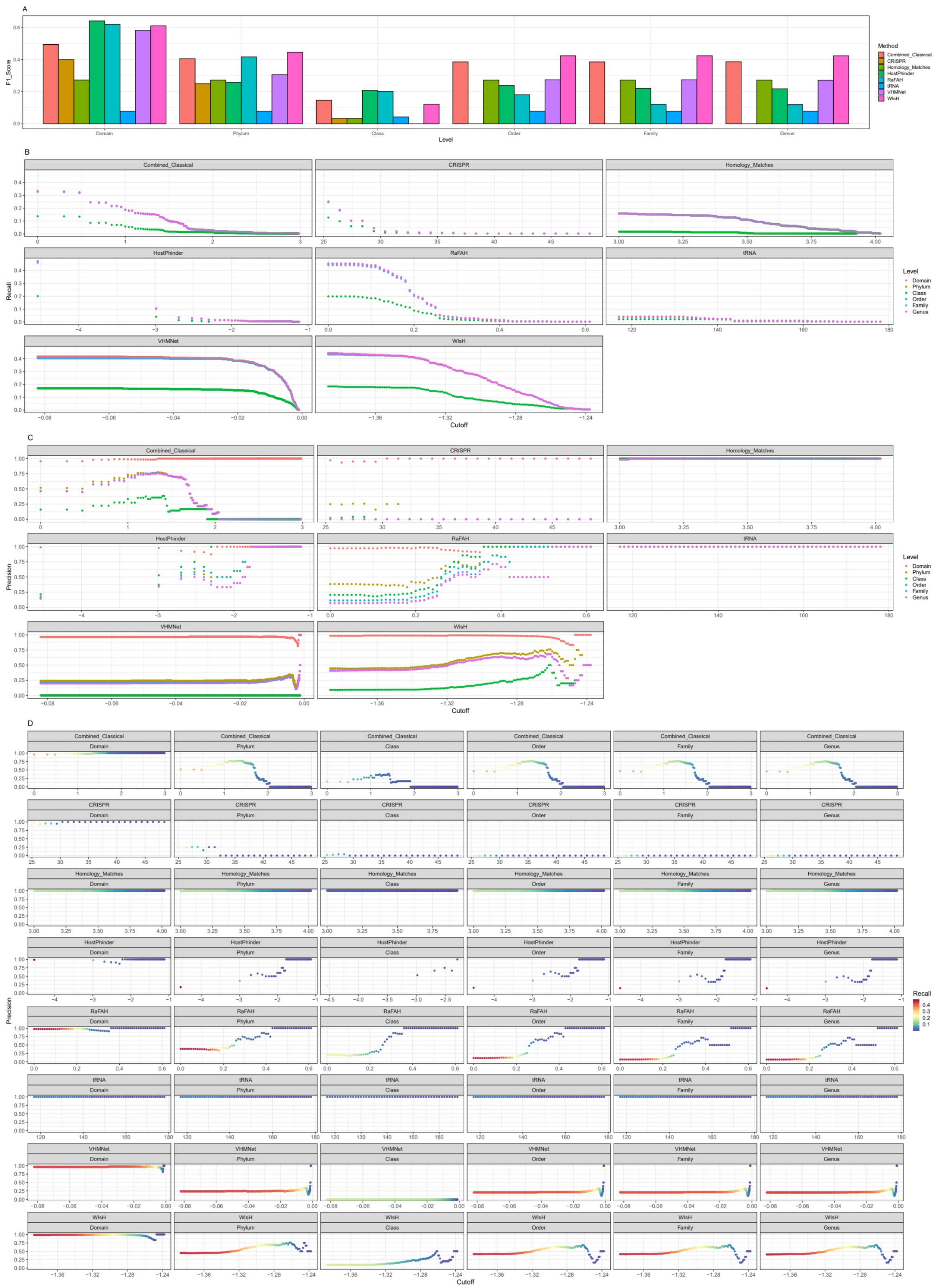

Figure S3: Performance of host prediction tools on Test Set 2: Associations between score cutoff, precision, and recall for RaFAH, the alignment-free (WIsH and HostPhinder), hybrid

(VirHostMatcher-Net), and classical (CRISPR, tRNA and homology matches) host prediction approaches. A) F1-score of methods when considering all predictions regardless of score at multiple taxonomic levels. B) Association between score cutoff and recall for each taxonomic level. C) Association between score cutoff and precision for each taxonomic level D) Associations between precision and recall in function of score cutoff. Figure S8 depicts the association between precision and score cutoff of VirHostMatcher-Net for score values above the 75th percentile.

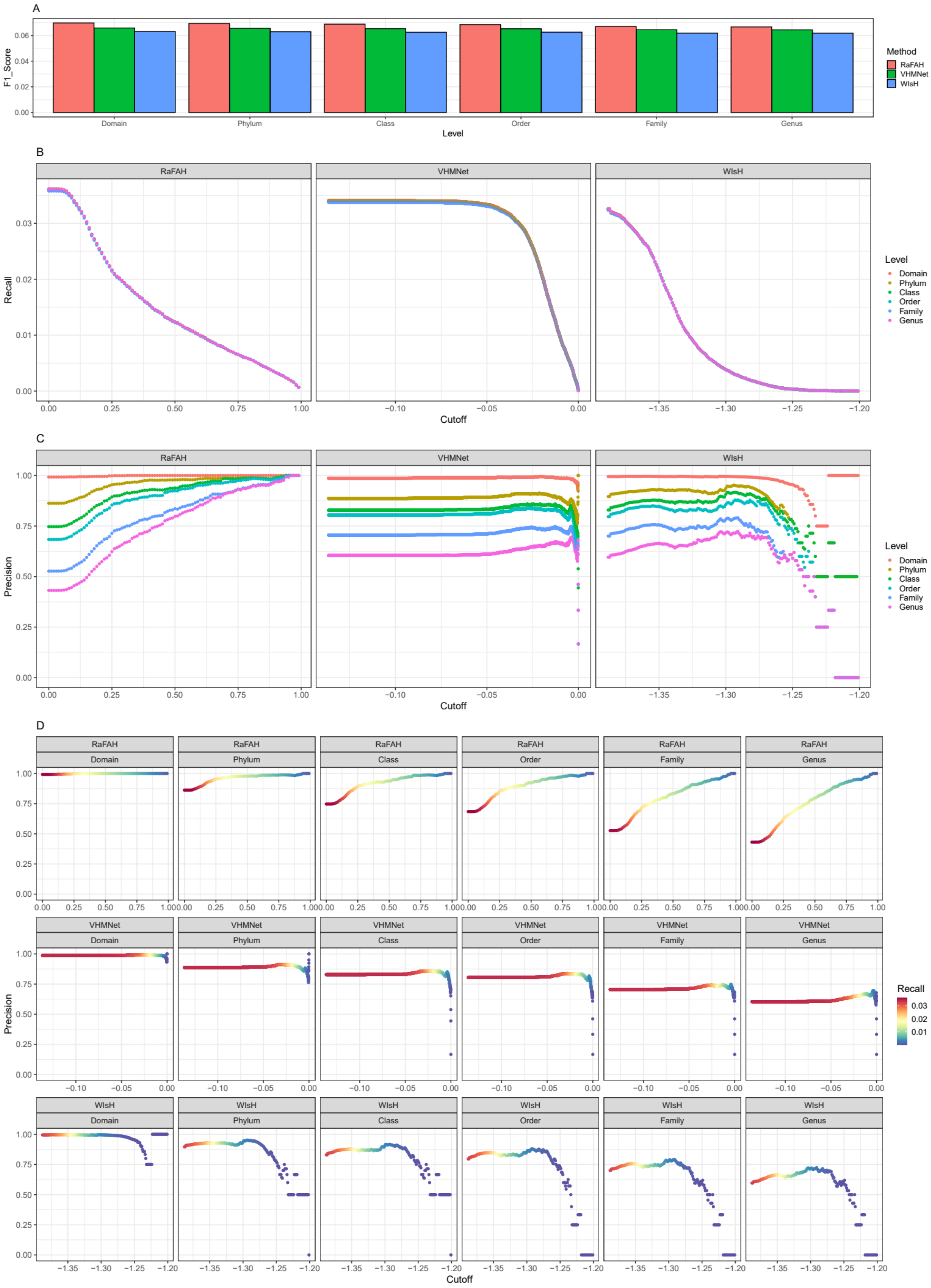

Figure S4: Performance of host prediction tools on Test Set 3: Associations between score cutoff, precision, and recall for RaFAH, WIsH and VirHostMatcher-Net. The hosts assigned by the

combined classical approaches were considered the true hosts of the genomes in Test Set 3. A) F1-score of methods when considering all predictions regardless of score at multiple taxonomic levels. B) Association between score cutoff and recall for each taxonomic level. C) Association between score cutoff and precision for each taxonomic level. D) Associations between precision and recall in function of score cutoff. Figure S8 depicts the association between precision and score cutoff of VirHostMatcher-Net for score values above the 75th percentile.

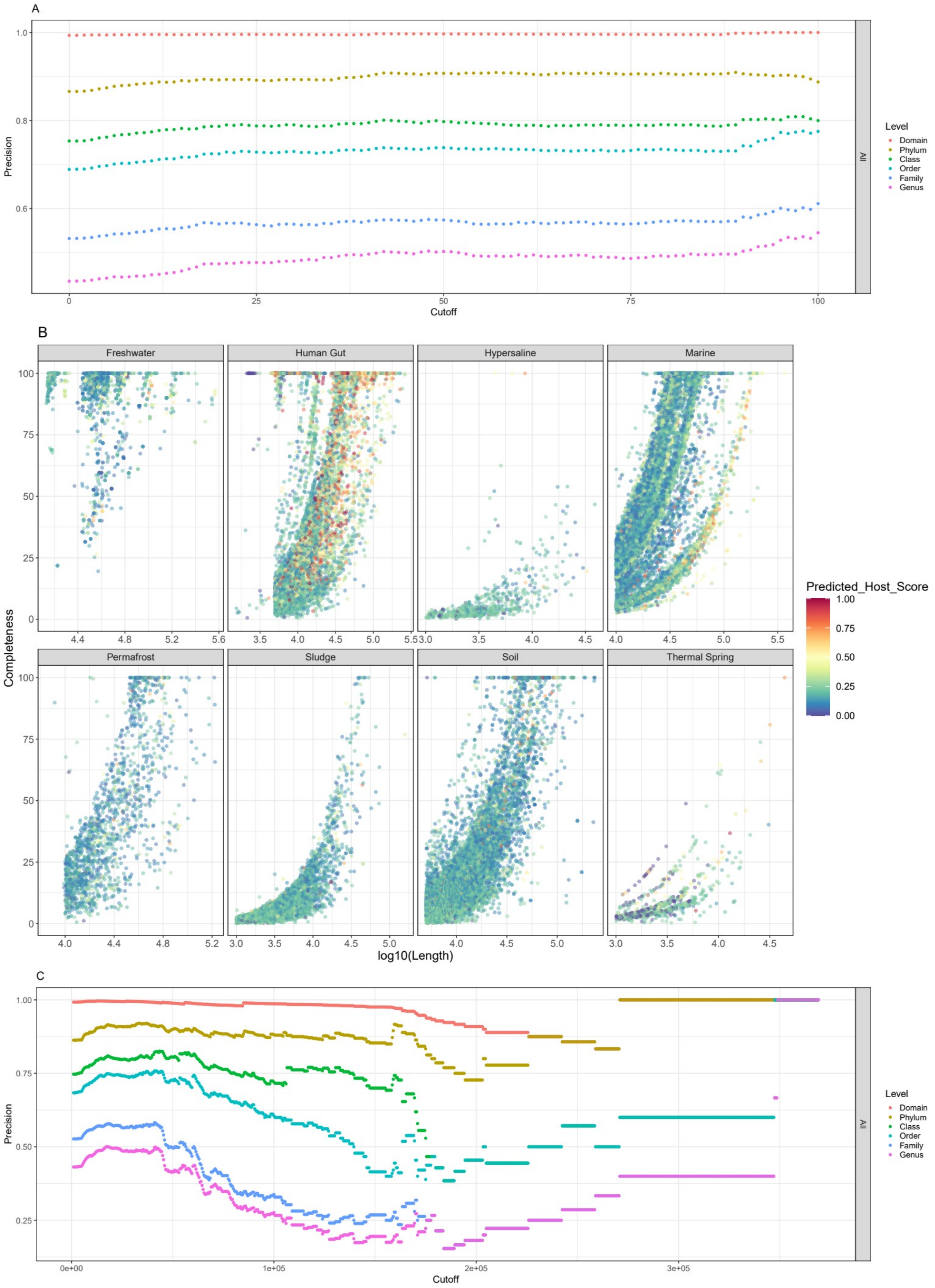

Figure S5: Associations between the performance of RaFAH and genome length/completeness on Test Set 3 genomes. A) Scatterplot displaying the cutoff for genome completeness (X axis) and

precision of RaFAH (y axis). B) Association between genome legngth (X axis) and genome completeness (Y axis) estimated with CheckV across 8 ecosystems (Panels). C) Scatterplot displaying the cutoff for genome length (X axis) and precision of RaFAH (y axis).

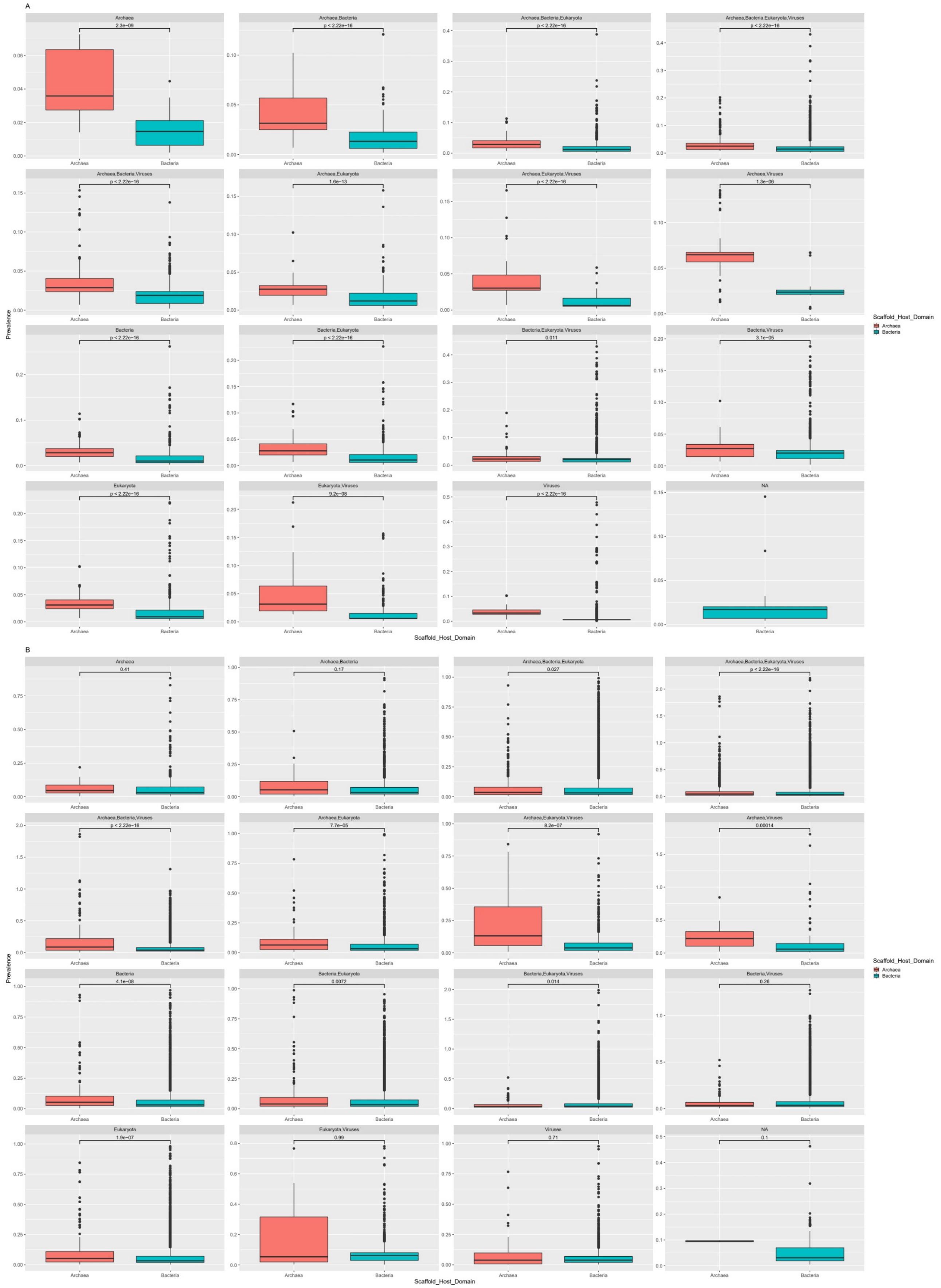

Figure S6: Prevalence of Pfam domains among viruses. Pfam domains were grouped according to their expected taxonomic ranges (depicted above each panel). Only values derived from scaffolds

with at least 5 CDS are shown to reduce noise. A) Comparisons of Pfam domain prevalence between RefSeq viruses of Archaea and Bacteria. The *p* values of each comparison obtained with the Mann-Whitney test are depicted above bars. B) Pfam domain prevalence between RefSeq viruses of Archaea and of Bacteria from TestSet3. Notice the different y axes on each panel.

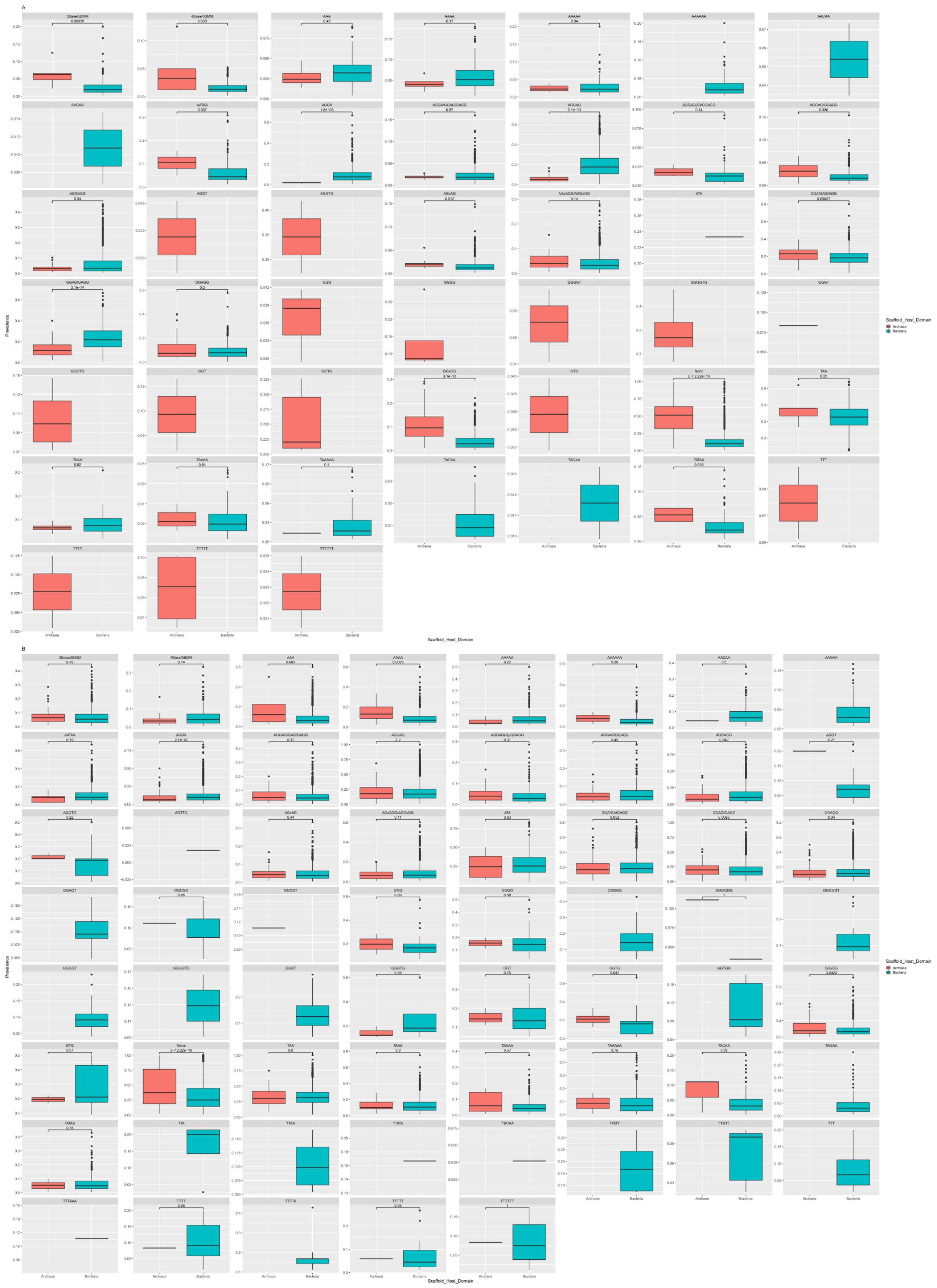

Figure S7: Prevalence of ribosomal binding site (RBS) motifs among viruses. Only values derived from scaffolds with at least 5 CDS are shown to reduce noise. A) Comparisons of RBS motif

prevalence between RefSeq viruses of Archaea and Bacteria. The *p*-values of each comparison obtained with the Mann-Whitney test are depicted above bars. B) Comparisons RBS motif prevalence between RefSeq viruses of Archaea and Bacteria and viruses from TestSet3.

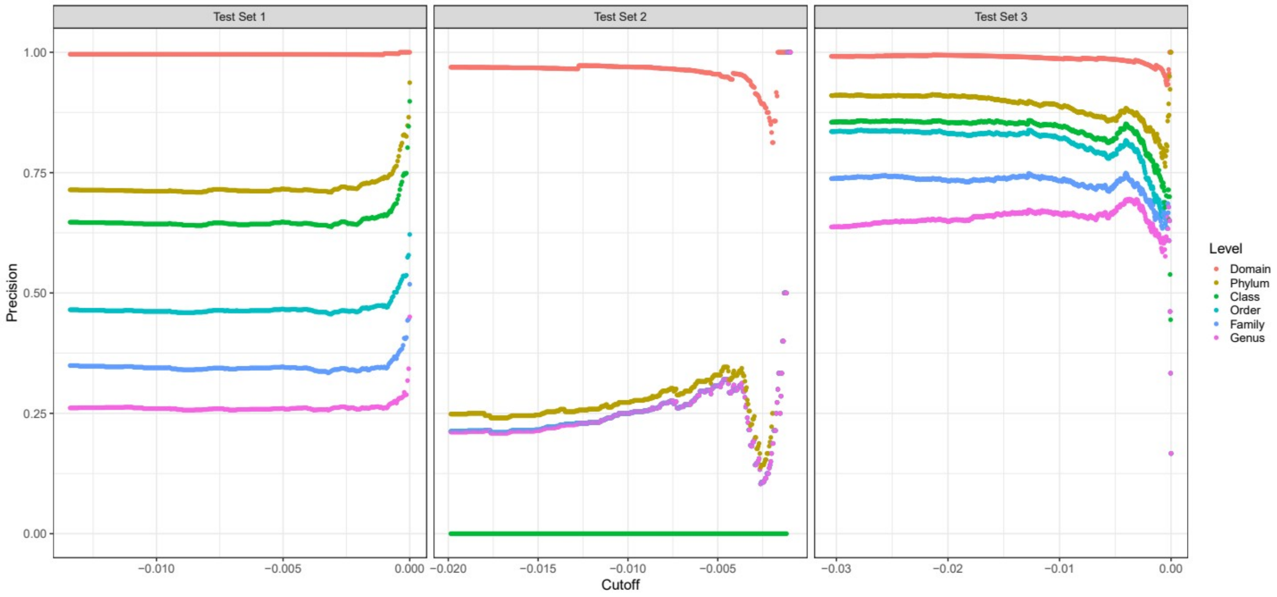

Figure S8: Association between precision and score cutoff for VirHostMatcher-Net in Test Sets 1, 2 and 3. All scores below the 75th percentile value were excluded from this analysis.

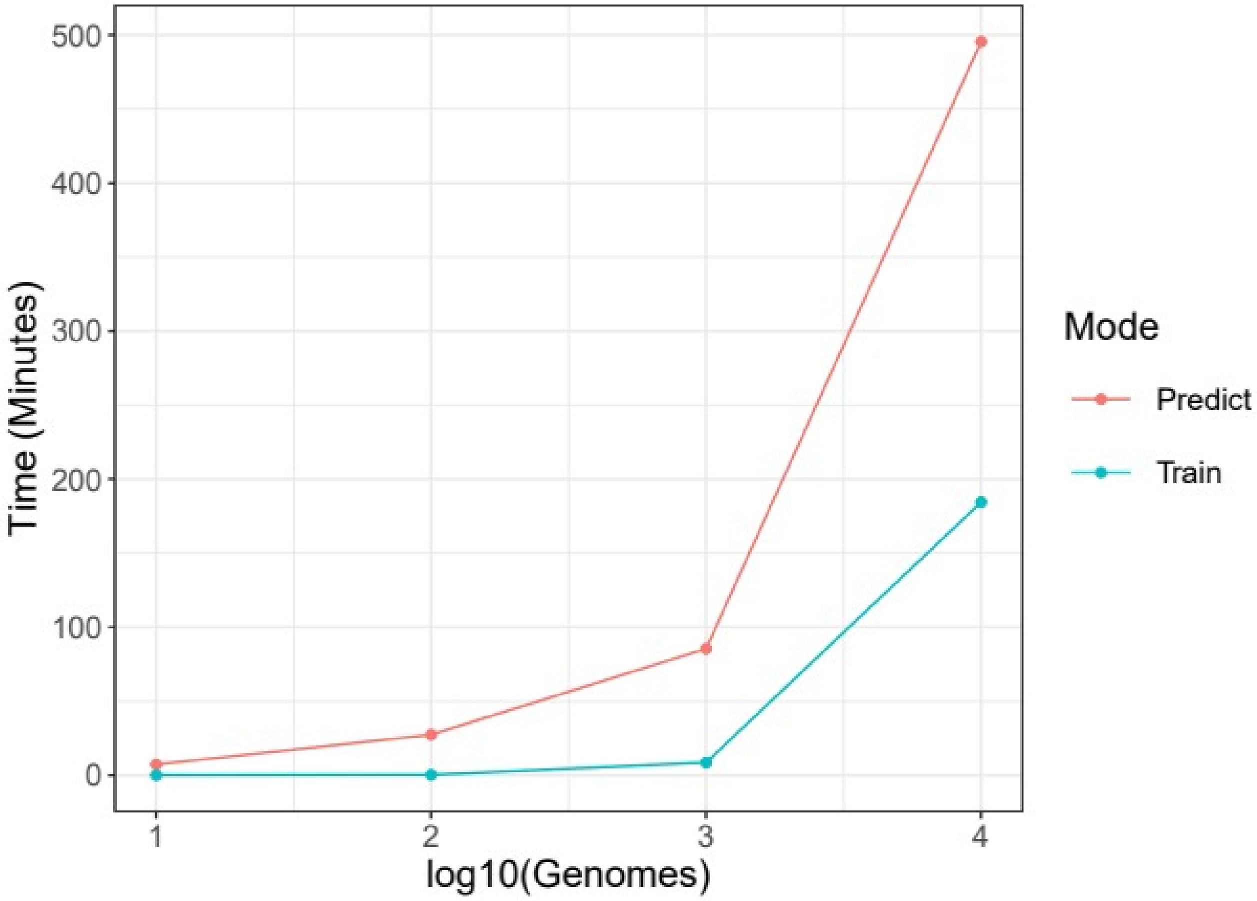

Figure S9: Timing of RaFAH computation on Training and Prediction modes (Y axis) as a function of the number of input genomes (X axis). Calculations were performed using randomly selected genomes of Test Set 3 on an Intel(R) Xeon(R) Gold 6140 CPU @ 2.30GHz machine using 24 threads.

Supplementary Data 1: Fasta file containing the nucleotide sequences from NCBI RefSeq and GLUVAB viruses that made up Training Sets 1, 2, and 3. Due to file size Supplementary Data 1 is available on Figshare with DOI: https://doi.org/10.6084/m9.figshare.14208500

Supplementary Data 2: Fasta file containing the nucleotide sequences from NCBI RefSeq that made up Testing Set 1. Due to file size Supplementary Data 2 is available on Figshare with DOI: https://doi.org/10.6084/m9.figshare.14210591

Supplementary Data 3: Fasta file containing the nucleotide sequences from SAG derived viruses that made up Testing Set 2. Due to file size Supplementary Data 3 is available on Figshare with DOI: https://doi.org/10.6084/m9.figshare.14208506

Supplementary Data 4: Fasta file containing the nucleotide sequences from metagenome derived viruses that made up Testing Set 3. Due to file size Supplementary Data 4 is available on Figshare with DOI: https://doi.org/10.6084/m9.figshare.14210612
