# Supplementary material for: RaFAH: Host prediction for viruses of Bacteria and Archaea based on protein content
Source: Patterns (N Y). 2021 Jun 15;2(7):100274. doi: 10.1016/j.patter.2021.100274 (PMC8276007; doi:10.1016/j.patter.2021.100274)
Supplement: Document S2. Article plus supplemental information [file mmc7.pdf]

Descriptor

Patterns

RaFAH: Host prediction for viruses of Bacteria and Archaea based on protein content

Highlights

- RaFAH was developed to predict the hosts of viruses of Bacteria and Archaea
- RaFAH displayed comparable or superior performance to other host-prediction tools
- RaFAH performed well across viromes from eight different ecosystems
- RaFAH identified hundreds of genomic sequences as derived from viruses of Archaea

Authors

Felipe Hernandes Coutinho,
Asier Zaragoza-Solas,
Mario López-Pérez, ..., Bas E. Dutilh,
Robert Edwards,
Francisco Rodriguez-Valera

Correspondence

fhernandes@icm.csic.es

In brief

We developed a machine-learning tool called RaFAH that uses genomic data to predict the hosts of viruses of Bacteria and Archaea. This tool led to an expansion of the catalog of known archaeal viruses and has potential to help better characterize the global virosphere by linking viruses to their hosts with high precision and recall.

Coutinho et al., 2021, Patterns 2, 100274
July 9, 2021 © 2021 The Authors.
https://doi.org/10.1016/j.patter.2021.100274

CellPress

Patterns

CellPress
OPEN ACCESS

Descriptor

RaFAH: Host prediction for viruses of Bacteria and Archaea based on protein content

Felipe Hernandes Coutinho,[1,8,*] Asier Zaragoza-Solas,[1] Mario López-Pérez,[1] Jakub Barylski,[2] Andrzej Zielezinski,[3] Bas E. Dutilh,[4,5] Robert Edwards,[6] and Francisco Rodriguez-Valera[1,7]
[1]Evolutionary Genomics Group, Departamento de Producción Vegetal y Microbiología, Universidad Miguel Hernández, Aptdo. 18., Ctra. Alicante-Valencia N-332, s/n, San Juan de Alicante, 03550 Alicante, Spain
[2]Molecular Virology Research Unit, Faculty of Biology, Adam Mickiewicz University Poznan, 61-614 Poznan, Poland
[3]Department of Computational Biology, Faculty of Biology, Adam Mickiewicz University Poznan, 61-614 Poznan, Poland
[4]Centre for Molecular and Biomolecular Informatics (CMBI), Radboud University Medical Centre/Radboud Institute for Molecular Life Sciences, 6525 GA Nijmegen, the Netherlands
[5]Theoretical Biology and Bioinformatics, Science for Life, Utrecht University (UU), 3584 CH Utrecht, the Netherlands
[6]College of Science and Engineering, Flinders University, Bedford Park, SA 5042, Australia
[7]Moscow Institute of Physics and Technology, Dolgoprudny 141701, Russia
[8]Lead contact
*Correspondence: fhernandes@icm.csic.es
https://doi.org/10.1016/j.patter.2021.100274

THE BIGGER PICTURE Viruses that infect Bacteria and Archaea are ubiquitous and extremely abundant. Recent advances have led to the discovery of many thousands of complete and partial genomes of these biological entities. Understanding the biology of these viruses and how they influence their ecosystems depends on knowing which hosts they infect. We developed a tool that uses data from complete or fragmented genomes to predict the hosts of viruses using a machine-learning approach. Our tool, RaFAH, displayed performance comparable with or superior to that of other host-prediction tools. In addition, it identified hundreds of sequences as derived from the genomes of viruses of Archaea, which are one of the least characterized fractions of the global virosphere.

1 2 3 4 5 **Proof-of-Concept:** Data science output has been formulated, implemented, and tested for one domain/problem

SUMMARY

Culture-independent approaches have recently shed light on the genomic diversity of viruses of prokaryotes. One fundamental question when trying to understand their ecological roles is: which host do they infect? To tackle this issue we developed a machine-learning approach named Random Forest Assignment of Hosts (RaFAH), that uses scores to 43,644 protein clusters to assign hosts to complete or fragmented genomes of viruses of Archaea and Bacteria. RaFAH displayed performance comparable with that of other methods for virus-host prediction in three different benchmarks encompassing viruses from RefSeq, single amplified genomes, and metagenomes. RaFAH was applied to assembled metagenomic datasets of uncultured viruses from eight different biomes of medical, biotechnological, and environmental relevance. Our analyses led to the identification of 537 sequences of archaeal viruses representing unknown lineages, whose genomes encode novel auxiliary metabolic genes, shedding light on how these viruses interfere with the host molecular machinery. RaFAH is available at https://sourceforge.net/projects/rafah/.

INTRODUCTION

Viruses that infect Bacteria and Archaea are the most abundant and diverse biological entities on Earth. Because of their sheer abundance, genomic diversity, and the fact that most viruses are only found in specific ecological niches, they remain elusive. Culture-independent techniques such as metagenomics[1] have been pivotal in the effort to describe viral biodiversity. Computational approaches have been developed to link these novel viruses to putative hosts[2] by identifying genomic signals that are indicative of a virus-host association.

This is an open access article under the CC BY license (http://creativecommons.org/licenses/by/4.0/).

First, alignment-free methods such as *k*-mer profiles use nucleotide composition to predict the host of a viral genome. Some viruses adapt their oligonucleotide composition to that of the host they infect, a process that may be driven by the adaptation of the codon usage to the translational machinery and tRNA pool available in the host cell, exchange of the genetic material, co-evolution of regulatory sequences, and/or an evasion of the host defense systems. Hence, by identifying the prokaryote genome with the highest significant similarity to a viral genome, tools that exploit *k*-mer profiles assume that prokaryote genome to be the host of the virus in question. Alignment-free methods (e.g., WIsH) show very high recall (i.e., percentage of viral genomes linked to a host) but usually have low precision (i.e., percentage of correct virus-host associations among the predicted virus-host associations), with reported host-prediction accuracy for genus-level predictions between 33% and 64% depending on the dataset.[2–4] Similarities in *k*-mer profiles between viruses can also be used for host prediction following the same rationale (e.g., HostPhinder).[5]

Second, there are alignment-dependent approaches to assess similarity between viral and prokaryote genomes. These methods assume that genetic information exchange between viral and prokaryote genomes is indicative of virus-host associations. Specific genetic fragments, although short, might be informative for this purpose, such as CRISPR spacers and tRNA genes, while longer matches such as whole genes or integrated prophages can also provide an indication of virus-host linkage.[2] Both aforementioned approaches are limited by the fact that they require the genome of the host to be present in the reference database. That host should contain an active CRISPR system whose array should contain a spacer targeting (a close relative of) the phage, allowing identification of a protospacer without too many mismatches. Alignment-dependent approaches also require that detectable genetic exchange has taken place between virus and host. Hybrid approaches leverage on information from both alignment-free and alignment-dependent approaches for host prediction (e.g., VirHostMatcher-Net).[6]

Third, the gene content of viral sequences can be investigated in search of specific marker genes that are indicative of the host, such as photosynthesis genes for cyanophages.[7] This low-throughput approach may have high precision, but usually the recall of such predictions is low and the procedure is extremely time-consuming.

All of these approaches have been used extensively in viral metagenomic studies to predict hosts to uncultured viruses.[1,7–9] An ideal tool for virus-host prediction should combine the precision of alignment-dependent methods and the recall of alignment-free approaches. Furthermore, it should be independent of host genomes so as not to be limited by database completeness biases. Previous studies have shown that random forest algorithms are suitable for classifying viruses according to their hosts[10] and that protein domains can be used to achieve accurate host predictions.[11,12] Based on these findings, we postulated that random forest classifiers could be applied to protein content to build a classifier based on identifying combinations of genes that are indicative of virus-host associations. Through this approach, we were able to design RaFAH (Random Forest Assignment of Hosts), a classifier that combined the precision of manual curation, the recall of alignment-free approaches, and the speed and flexibility of machine learning (Figure 1).

RESULTS AND DISCUSSION

We tested the performance of RaFAH and other host-prediction approaches on an independent dataset of isolated viral genomes that did not overlap with those used for training the models (Test Set 1, composed of RefSeq viral genomes with less than 70% average amino acid identity when compared with those in Training Set 3, see experimental procedures). When using RaFAH and the other tested methods without score or prediction probability cutoff (i.e., considering as valid all host predictions with no thresholds for their probability value or bit score), RaFAH outperformed alignment-independent, hybrid, and alignment-dependent approaches for host prediction at every taxonomic level based on the F1 score (Figure 2A). This difference in performance became gradually more evident from domain to genus level. Next, we evaluated how the performance of these tools responded to thresholding (i.e., applying a cutoff on their probability value or bit score) and only considering predictions that were above the cutoffs. These analyses revealed that homology matches, CRISPR, tRNA, and combined classical approaches (i.e., homology matches, CRISPR, and tRNA, see experimental procedures) displayed the lowest recall (Figure 2B) but the highest precision (Figure 2C). HostPhinder and CRISPR displayed high precision only at the strictest score cutoffs. As a consequence, these two methods displayed very low recall when the highest cutoffs for predictions were established. RaFAH, WIsH, and VirHostMatcher-Net displayed higher recall than the other approaches, especially at the range of more permissive score cutoffs (0). Yet this higher recall came at the expense of lower precision for WIsH and VirHostMatcher-Net. Meanwhile the precision of RaFAH outperformed these tools even when no cutoffs were applied. Together, precision, recall, and F1 score suggest that RaFAH can predict more virus-host interactions than the other tested approaches while maintaining high precision, particularly for divergent viral genomes that escape detection by the classical approaches (Figure S1).

We evaluated how the similarity among the genomes in Test Set 1 with those used to train the model (Training Set 3) affected the performance of RaFAH. For this purpose, we assessed how the precision of RaFAH changed by setting a threshold on the maximum allowed average amino acid identity (AAI) between the genomes on Test Set 1 and those on Training Set 3. As expected, a positive association was observed between these variables (Figure S2), meaning that the more similar the testing genomes are to the ones used for training, the more likely RaFAH is to correctly predict their hosts at all taxonomic levels. Based on this analysis, 75% of the class-level host predictions will be correct (precision: ~0.75) for viruses that possess <60% AAI to the ones in the database, when no cutoffs on prediction score are applied.

We applied importance analysis to determine which protein clusters were most relevant for predicting viral hosts using RaFAH. The most important predictor was annotated as an Rz-like phage lysis protein (Table S1). Among the protein clusters that ranked among the 50 most important were multiple lysins, tail, and tail fiber proteins. These proteins are known to determine virus-host range, as they play fundamental roles in virus

CellPress
OPEN ACCESS

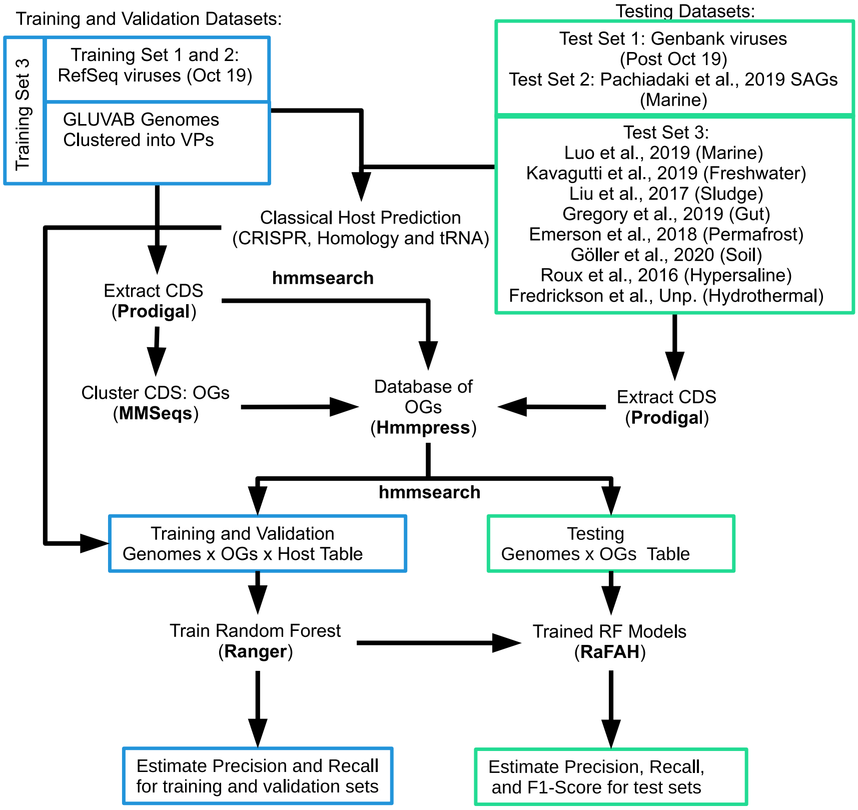

Figure 1. Overview of the strategy used to train, validate, and test random forest models

The training and validation sets were composed of viral RefSeq genome sequences published until October 2019 and viral genomic sequences derived from GLUVAB. GLUVAB sequences were clustered into viral populations (VPs) and assigned putative hosts through classical approaches (tRNA, homology matches, and CRISPR) on a per-population basis. Coding DNA sequences (CDS) were extracted from these sets and clustered into orthologous groups (OGs), aligned, and pressed into a database of hidden Markov model (HMM) profiles. Next, CDS were queried against this database to compute the bit scores of each CDS against each HMM, from which a matrix of Genomes × OG scores was derived. This matrix was used to train the random forest model. The performance of the model was evaluated on the training and validation sets according to precision and recall. The test sets comprised viral RefSeq genomes published after October 2019 (Test Set 1), viral genome fragments retrieved from marine SAGs (Test Set 2), and metagenomes/viromes from eight distinct ecosystems (Test Set 3). Similarly, CDS were extracted from these sets and queried against the HMM database derived from the training set to compute the bit scores of each CDS against each HMM, from which the testing matrix of Genomes × OG scores was derived and analyzed through RaFAH. From these results, the precision, recall, and F1 score of RaFAH were evaluated on the Test Sets.

entry and exit and host recognition.[13] The fact that these proteins ranked among the most important for RaFAH predictions is evidence that it learned to predict virus-host associations based on proteins that are directly involved in virus-host molecular interactions.

Host-prediction tools were further validated on a dataset of viral genomic sequences derived from marine single amplified genomes (SAGs), Test Set 2.[14] These sequences represent an ideal test dataset because they are uncultured viruses, not represented in the National Center for Biotechnology Information (NCBI) database used for training, and can confidently be assigned hosts because these viruses were inside or attached to the host cells during sample processing. Based on the F1 score, HostPhinder displayed the best performance at the levels of domain and class, followed by RaFAH slightly behind (Figure S3A). Yet at the level of phylum WIsH displayed the best performance, again followed closely by RaFAH. At the levels of order, family, and genus, WIsH displayed the highest F1 scores followed by the combined classical approaches. The recall (Figure S3B) and precision (Figure S3C) of RaFAH on Test Set 2 was lower than that obtained for Test Set 1. Nevertheless, a negative association between precision and recall as a function of the score cutoff was also observed for RaFAH and the other tested tools on Test Set 2 (Figure S3D). Taken together, these results are evidence that RaFAH also performed well when predicting hosts of uncultured viruses from the marine ecosystem.

Some features of Test Set 2 must be considered when interpreting these results. First, most of the viruses identified in Test Set 2 were derived from single-cell genomes classified as either *Pelagibacter*, *Puniceispirillum*, *Prochlorococcus*, and *Synechococcus*. This is expected considering these are the most abundant organisms at the ecosystem from which this dataset is derived. Nevertheless, this relatively low diversity of taxa has implications for the assessment of host-prediction tools. For instance, the genera *Prochlorococcus* and *Synechococcus* have no determined taxonomy at the level of class. Therefore, predictions at this level do not count toward precision for these particular taxa. As a consequence, the precision of all host-prediction tools displayed a steep decrease at this taxonomic level. This was particularly noticeable for VHM-Net for which all correct predictions were restricted to the two aforementioned taxa, which led to 0% precision at the level of class. Second, the majority of bacteriophage genomes in Test Set 2 have very low completeness (median 6.85%, estimated by CheckV,[15] see experimental procedures). The low diversity of hosts and the very low genome completeness (as evaluated below) likely impacted the performance of RaFAH on this dataset. Third, because most viral genomes in Test Set 2 belong to four genera, RaFAH is likely to have its performance hindered due to the number of phage genomes that infect these genera available in Training Set 3. Meanwhile, approaches that rely on host genomes are likely to be hindered by the number of these genomes available in the reference database.

To test the performance of RaFAH on samples from other habitats, we applied it to predict hosts of a dataset of viral genomes obtained from metagenomes of eight different ecosystems (Test Set 3). For comparison, we also applied the other tested methods of host prediction (HostPhinder did not scale to the more than

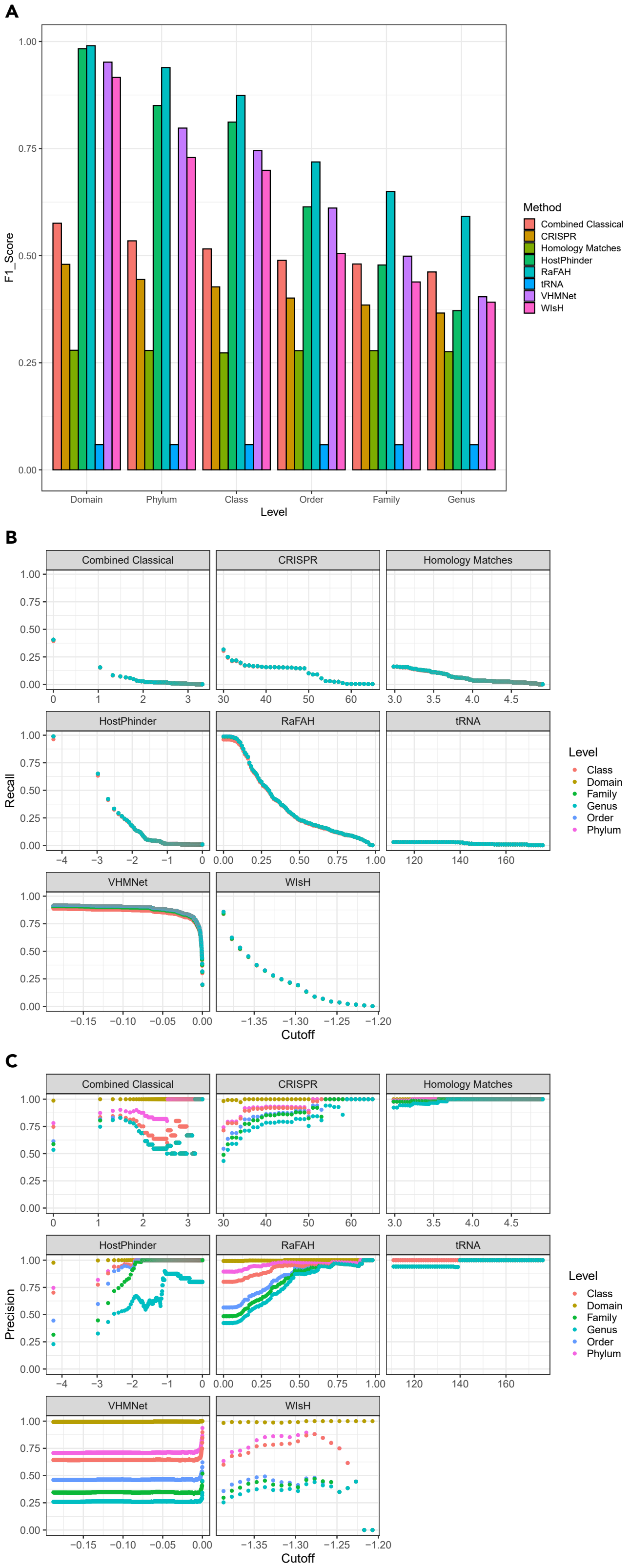

60,000 genomes in this dataset, and analyses did not complete after running for several days). According to the F1 score, RaFAH outperformed WIsH and VirHostMatcher-Net for this dataset as well (Figure S4A), due to slightly higher recall (Figure S4B) and precision (Figure S4C). RaFAH was also superior when the strictest cutoffs were applied, whereby both precision and recall were markedly superior to VirHostMatcher-Net (Figure S4D). On this dataset, RaFAH achieved 43.13% precision at the level of genus when no score threshold was applied. Bootstrap analysis revealed that this level of precision was consistent across 1,000 replicates (mean 43.02% ± 2.1%). This result indicates that the precision of RaFAH on Test Set 3 was not biased by uneven viral genome diversity among the samples that made up this dataset.

When using classical approaches for host prediction, the majority of viruses remained unassigned regardless of ecosystem, and the best performance of these approaches was among the human gut dataset, in which only about 25% of sequences (lengthwise) could be assigned to a host at the level of phylum (Figure 3). Meanwhile, when set to the 0.14 cutoff, which yielded 92% phylum level precision on Test Set 1 (Figure S1) and 90% on Test Set 3 (Figure S4D), RaFAH was capable of predicting putative hosts to the majority of viral sequences across all ecosystems except for the permafrost dataset, likely because viruses derived from this ecosystem are poorly represented in reference databases.

Interestingly, the host predictions yielded by RaFAH were markedly different across ecosystems. Viruses of Proteobacteria were the dominant group in all ecosystems except the human gut. As expected, the most abundant targeted hosts of the viruses from each ecosystem were the most abundant taxa that reside in those habitats. Viruses of Cyanobacteria were the second most abundant group among the marine dataset, a position that was occupied by viruses of Actinobacteria and Bacteroidetes among the freshwater dataset. Viruses of Firmicutes and Bacteroidetes were the dominant group among the dataset of human gut viruses while viruses of Firmicutes, Bacteroidetes, and Actinobacteria were among the most abundant among the soil and permafrost datasets. Viruses of Euryarchaeota were the second most abundant group among the hypersaline dataset, a position that was occupied by viruses of Crenarcheaota in the thermal springs dataset. These results are in accordance with the known prokaryote diversity that dwells in each of these ecosystems.[8,16–22] Although this agreement between virus and host community composition is to be expected, it is seldom observed in studies of viral ecology based on metagenomics because classical methods leave the majority of viruses without host predictions. RaFAH circumvents these issues by providing an accurate and complete description of viral communities regarding their targeted hosts.

Figure 2. Performance of RaFAH compared with alignment-free and classical host-prediction approaches on Test Set 1

(A) F1 score of methods when considering all predictions regardless of score at multiple taxonomic levels.

(B) Association between score cutoff and recall of predictions for each method.

(C) Association between score cutoff and precision of predictions for each method. The score cutoffs for HostPhinder, Homology matches, VirHostMatcher-Net, and combined classical are shown on the $\log_{10}$ scale. Figure S8 depicts the association between precision and score cutoff of VirHostMatcher-Net for score values above the 75th percentile.

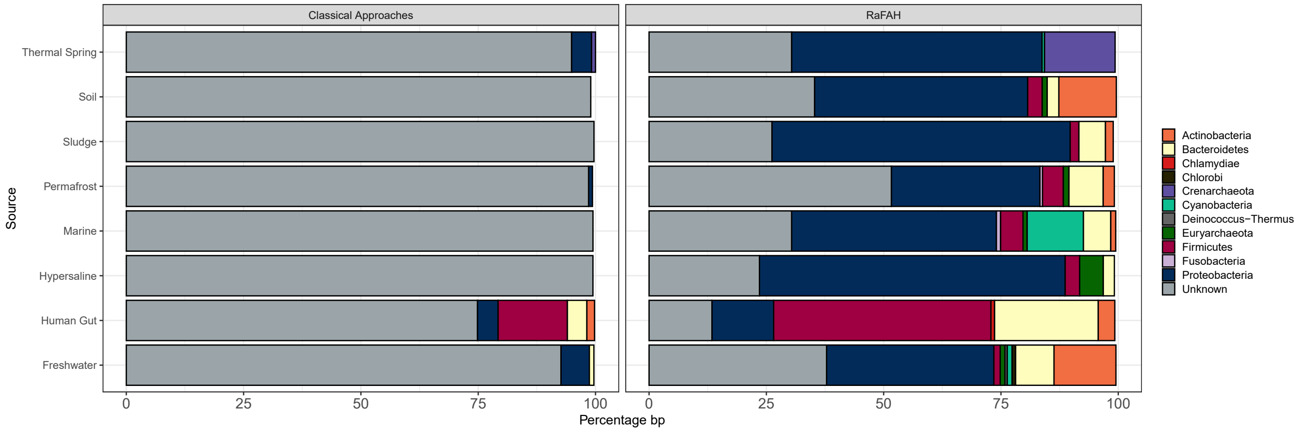

Figure 3. Description of the viromes of eight ecosystems using combined classical host-prediction approaches and RaFaH

For each dataset we calculated the fraction of the assembly predicted to each putative host phylum by each method. Phyla that represent less than 0.5% of the total assembly are not shown.

We assessed how genome completeness affected the performance of RaFAH. For this purpose, we used Test Set 3 as it displayed the necessary range of genome completeness values necessary for this purpose, while Test Set 1 was mostly made up of complete genomes and Test Set 2 was mostly made up of low-completeness genomes. We assumed that the predictions yielded by the combined classical approaches represented the true hosts of Test Set 3, although this assumption is likely to lead to an underestimation of the true precision of RaFAH. We found weak positive associations (Pearson $R^2 > 0.6$, $p < 10^{-13}$ for all taxonomic levels) between the precision of RaFAH and genome completeness at all taxonomic levels (Figure S5A). These curves tended to reach a plateau around ~25%–50% genome completeness and increased further for the lower taxonomic ranks (genus, family, and order) for genomes that were >85% complete. Coupled with the observations of the performance of RaFAH on Test Set 2, we suggest that RaFAH is better suited for viral genomes with 50% or more completeness. We used Test Set 3 to analyze the relationship between genome completeness, sequence length, and RaFAH prediction score across the eight different ecosystems (Figure S5B). This revealed a positive correlation between those variables (Pearson R^2 = 0.65, $p < 2.2e-16$ for the combined set of all ecosystems). Likewise, significant albeit weaker positive correlations were also detected between prediction score and sequence length (Pearson R^2 = 0.14, $p < 2.2e-16$), and prediction score and genome completeness (Pearson R^2 = 0.11, $p < 2.2e-16$). We found that regardless of taxonomic level, precision did not consistently increase through thresholding for genome length, providing further evidence that shorter sequences do not necessarily yield worst predictions (and vice versa) (Figure S5C). These results suggest that the precision of RaFAH cannot be explained by genome length/completeness alone, likely because RaFAH was trained on a dataset with a majority of genome fragments.

We also performed analysis of the combined effects of the relevant variables and how those, together, affected precision, recall, and the F1 score of RaFAH using Test Set 3. Taken together, these results demonstrated that the performance of RaFAH on a given genome is dependent on each of ecosystem source, genome completeness, similarity of the genome to those in the training dataset, and the taxonomic level being considered (see Table S6 at https://doi.org/10.6084/m9.figshare.14365562). For this reason, there is not a single score threshold that is ideal for all use cases. Nevertheless, we make the following recommendations. For differentiating between viruses of Bacteria and Archaea, RaFAH has nearly 100% precision even at the most permissive cutoff (0), thus for this particular purpose it can be applied without threshold. For a broad characterization of multiple viral genomes from an ecosystem, permissive thresholds are acceptable. For example, to compare viral host prevalences across different metagenomes at the level of phylum, we recommend a threshold of 0.14. This yields a precision of approximately 90% without sacrificing recall (Figures S1 and S4D), regardless of ecosystem source, genome length, completeness, or similarity to the training dataset. At lower taxonomic levels, stricter cutoffs are necessary. Users can select cutoffs according to the desired precision based on the curves depicted in Figures S1 and S4D. As a rule, longer, more complete genomes with higher maximum AAI values to genomes in the test set should allow more permissive cutoffs.

Based on the finding that RaFaH achieved nearly perfect precision for domain-level host predictions, and the fact that viruses of Archaea are under-represented in databases, we subsequently focused on the description of these viruses. Few large-scale studies have addressed the diversity of uncultured viruses of Archaea, and they focused mostly on marine samples.[23–26] Here, we describe viruses from seven other ecosystems: soil, permafrost, freshwater, sludge, hypersaline lakes, thermal springs, and the human gut. Applying RaFAH to only eight metagenomic datasets led to the prediction that 537 genomic sequences represent viruses of Archaea (prediction score ≥ 0.14). To put this figure in context, there are only 96 genomes of viruses of Archaea deposited in the NCBI RefSeq database.

We took several steps to ensure that these genomes were truly derived from viruses of Archaea and consistently found compelling evidence to support our claim. First, these genomes could be linked to archaeal genomes either through homology matches or alignment-independent approaches, which provided further evidence that 423 out of the 537 genomes (79%) were indeed

derived from archaeal viruses (Table S2). Second, much like the RefSeq genomes of archaeal viruses, these sequences were enriched in Pfam domains annotated as exclusive of Archaea, eukaryotes, and their viruses (Figure S6). Third, these genomes were enriched in ribosomal binding site motifs that are also enriched among RefSeq viruses of Archaea (Figure S7).

Next, we manually inspected the gene content of the viruses predicted to infect Archaea in search of novel auxiliary metabolic genes (AMGs) and new mechanisms of interaction with the host molecular machinery. The small number of reference genomes of Archaea and their viruses makes it difficult to describe the gene content of the archaeal viruses that we discovered because most of their genes have no taxonomic or functional annotation. However, we found several sequences containing genes coding for thermosomes, group II chaperonins involved in the correct folding of proteins, homologous to their bacterial counterparts, GroEL/GroES.[27] Other AMGs found among archaeal viruses were those involved in the synthesis of cobalamin *cob*S, recently associated with Marine Group I (MGI) Thaumarchaeota virus infection[26] as well as genes that encoded 7-cyano-7-deazaguanine synthase QueC involved in archaeosine tRNA modification.[28] One of the AMGs most prevalent among archaeal viral genomes encoded for a molybdopterin biosynthesis MoeB protein (ThiF family). This family of proteins is involved in the first of the three steps that make up the ubiquitination process.[29] This system regulates several cellular processes through post-translational modification of proteins such as their function, location, and degradation, making it an ideal target from the point of view of viruses to facilitate their replication.[30]

In conclusion, we developed a new tool that uses a random forest classifier based on protein content for virus-host prediction with great potential for studies of viral biodiversity and ecology. RaFAH frequently outperformed other methods that we tested and displayed high accuracy and recall in a dataset of cultured viruses, which extended to uncultured viruses from a diverse set of ecosystems. By analyzing metagenomic datasets from eight different ecosystems, RaFAH allowed for a significant expansion of the archaeal virosphere and shed light on their yet poorly understood content of AMGs. Future studies will describe even more uncultured viral sequences, and RaFAH will likely play a role on describing their hosts and allowing us to decipher their ecological roles. The addition of new viruses with predicted or experimentally verified hosts will allow RaFAH to evolve to identify viruses for an even larger diversity of hosts, and possibly at deeper taxonomic levels such as species. Likewise, these advancements will likely contribute to increasing the accuracy of RaFAH at all taxonomic levels.

EXPERIMENTAL PROCEDURES

Resource availability

Lead contact

Felipe Hernandes Coutinho, fhernandes@icm.csic.es.

Materials availability

This study did not generate new unique materials or reagents.

Data and code availability

All the data (viral and prokaryote genomes) analyzed in this study are freely available from public repositories. The data were also made available as part of the supplemental information. RaFAH and the associated files necessary to run it are freely available online at https://sourceforge.net/projects/rafah/. In addition, we created a Docker container with all the necessary dependencies, scripts, and files available at https://hub.docker.com/r/fhcoutinho/rafah.

Viral genomes database for model training and validation

Two datasets of viral genomes were used for both training and validating the random forest models. The first dataset contained the genomes of viruses of Bacteria and Archaea from NCBI RefSeq available on October 2019, which comprised 2,668 genomes along with their associated host data (Table S3). To avoid overestimating precision due to identical and nearly identical genomes in the database, this dataset was made non-redundant using CD-HIT[31] at a clustering cutoff of 95% identity over 50% alignment of the shorter sequence. The second dataset comprised the 195,698 GLUVAB genomes.[32] GLUVAB is a database of uncultured viral genomes compiled from multiple studies that covered several ecosystems. Only those sequences classified as bona fide viruses of prokaryotes in the original publication were used in subsequent analysis (Table S4).

Classical host prediction for GLUVAB genomes

To use GLUVAB genomes for training and validation of the random forest models, we first had to assign them to putative hosts using classical approaches. To minimize errors during this step we opted for using only alignment-dependent methods due to their higher precision.[2] The RefSeq genomes of Bacteria and Archaea were used as the reference database. We used three lines of evidence for virus-host associations: CRISPR spacers, homology matches, and shared tRNAs. CRISPR spacers were identified in the RefSeq genomes as previously described.[33] The obtained spacers were queried against the sequences of bona fide viral sequences using BLASTn v2.6.0+ (task blastn-short). The cutoffs defined for these searches were minimum identity of 100%, minimum query coverage of 100%, with no mismatches and maximum e-value of 1. Homology matches were performed by querying viral sequences against the databases of prokaryote genomes using BLASTn.[34] The cutoffs defined for these searches were minimum alignment length of 500 bp, minimum identity of 95%, and maximum e-value of 0.001. tRNAs were identified in viral scaffolds using tRNAScan-SE v1.2 [35] using the bacterial models. The obtained viral tRNAs were queried against the RefSeq database of prokaryote genomes using BLASTn. The cutoffs defined for these searches were minimum alignment length of 60 bp, minimum identity of 97%, minimum query coverage of 95%, maximum of 10 mismatches, and maximum e-value of 0.001. These steps for host assignment did not include the prophages in the GLUVAB database, as we were already confident of their host assignments.

We developed a per-viral population scoring method. First, all GLUVAB genomes were clustered into viral populations (VPs) on the basis of 95% average nucleotide identity and 80% shared genes.[36] For each virus-taxon association signal detected (i.e., homology, tRNA, or CRISPR), 3 points were added to the taxon if it was a CRISPR match, 2 points if it was a homology match, and 1 point if it was a shared tRNA. The taxon that displayed the highest score was defined as the host of the viral population. With this approach we ensured that all the genomes in the same VP were assigned to the same host and that no sequences had to be excluded due to ambiguous predictions.

Protein cluster inference and annotation

Protein sequences were identified in viral genomes using Prodigal[37] in metagenomic mode. Hidden Markov models (HMMs) for the phage proteins were built as follows. The 4,701,074 identified proteins were clustered by the cluster workflow of the MMseqs2 software suite,[38] with parameters: 35% sequence identity and alignment coverage had to cover at least 70% of both proteins. Protein clusters (PCs) were aligned into multiple sequence alignments (MSAs) using QuickProbs[39] with default parameters, then converted into HMMs using the hmmake program from the HMMER suite,[40] which resulted in 144,613 HMMs. The HMM profiles were annotated by performing HMM-to-HMM annotation against the pVOG database[41] using the HH-suite3 software suite.[42] First, the MSAs provided on the pVOGs website and the ones built in the previous step were converted into the hhsuite proprietary HMM format using hhmake. The pVOG HHMs were built into an HH-suite3 database, which was then used to find matches to the phage protein HMMs using hhsearch. All HMMs could be annotated through this approach, but only 4,578 matches displayed target coverage $\geq$50% and e-value $\leq 1^{-10}$.

Finally, individual viral proteins were mapped to the HMM profiles using the hmmsearch program limiting hits to those with e-value $\leq 10^{-5}$, alignment length $\geq$70% for both proteins, and minimum score of 50. These results were parsed into a matrix of viral genomes × PCs in which the values of each cell corresponded to the bit score of the best hit of each protein to a given PC, or zero if the protein and the PC did not match or if the score of the match was below the aforementioned 50 cutoff. Once the matrix of genomes × PC was defined, we calculated Pearson correlation coefficients (r) between all possible pairwise combinations of PCs. To remove redundancies, we grouped PCs into superclusters if they presented $r \geq 0.9$, and only a single PC from each supercluster was kept for subsequent analysis. This reduced table of genomes versus PC scores (25,879 genomes × 43,644 PCs) was used as input to train, validate, and test the random forest models.

Random forest training, validation, and testing

Our rationale was that the machine could learn the associations between genes and hosts much more efficiently than a human while also using the information contained in the hypothetical proteins. Hence, random forest models were built using the Ranger[43] package in R.[44] The response variable was the genus-level host assignment of the viral sequences while the input parameters were the scores of viral genomes to each PC. Multi-class random forests were built with 1,000 trees, 5,000 variables to possibly split at in each node, and using probabilistic mode. This classification approach ensured that a single model could be used for all virus genomes. The putative host of a viral genome was selected as the taxon with the highest probability score yielded by the random forest. The taxonomic classification of each genus up to the domain level was obtained by parsing the NCBI Taxonomy database with a custom script. Next, variable importance was estimated using the impurity method. When training the models and reporting predictions, we assumed that a virus can only infect a single genus. Due to the probabilistic nature of the random forests, all genera are associated with a score (which ranges from 0 to 1). Users interested in multi-genera viruses can search for those genomes that have close or equal scores as preliminary evidence that the viral genome in question might infect across multiple genera.

Three models were built and validated on independent datasets. Model 1 was trained on Training Set 1, which comprised 80% randomly selected non-redundant viral genomes from NCBI RefSeq. The performance of this model was evaluated on Training Set 1 and Validation Set 1, which comprised the remaining 20% of non-redundant RefSeq genomes. This process was repeated for a 10-fold cross-validation. Even without thresholding, these models exhibited high precision for both the training (mean 99.96% ± 0.026%) and validation sets (mean 76.47% ± 1.523%) at the genus level. Model 2 was trained on Training Set 2, which comprised 100% of the RefSeq genomes, and validated on Validation Set 2, which was comprised of GLUVAB genomes that could be assigned to a host at the level of genus by the pipeline described above. Finally, Model 3 was built based on Training Set 3, which comprised all of the RefSeq viral genomes and the GLUVAB genomes that could be assigned to a host at the level of genus (i.e., a combination of Training Set 2 and Validation Set 2). In this dataset each genus was represented by a median of three genomes, and for 187 out of 617 (30.3%) genera the model was trained with a single genome (Table S5). Models 1 and 2 were used as proof-of-principle models, and Model 3 was the definitive model used for testing and which is provided to the users and used for all subsequent analyses.

Viral genome completeness is likely to influence the performance of the models. A tool trained solely on complete or nearly complete genomes might not be capable of producing accurate predictions for the genome fragments that are often obtained with metagenomic datasets. Completeness of the 25,879 sequences used to train RaFAH was estimated with CheckV,[15] which indicated that this dataset encompassed both complete viral genomes as well as partial viral contigs. Partial viral genomes were the majority of sequences used to train RaFAH. Altogether, the genomes used for training displayed an average completeness of 53.6% ± 32.3%. According to CheckV, these sequences were classified as complete genomes (709 sequences), high-quality genome fragments (5,823), medium-quality genome fragments (5,493), low-quality genome fragments (13,707) and not determined (147).

We used three independent test sets to evaluate the performance of RaFAH Model 3. Test Set 1 comprised viral genomes retrieved from NCBI Genomes database in January 2021. We took several steps to make sure that Test Set 1 represented a challenging dataset for the random forest model so as to assess its ability to extrapolate. First, we excluded from Test Set 1 any genomes made public before November 2019. Second, Test Set 1 was made non-redundant at 95% nucleotide identity and 50% alignment length of the shorter sequence. Third, protein sequences derived from Test Set 1 were compared with the protein sequences of Training Set 3 using DIAMOND.[45] Any genomes that shared more than 70% of proteins or more than 70% average AAI with any genome from Training Set 3 were removed from Test Set 1. These steps resulted in an independent Test Set 1 consisting of 561 (out of the initial 3,427) genomes with no overlap to the genomes used to train the models.

Test Set 2 comprised viral genomes identified in SAGs from marine samples.[14] A total of 4,751 SAGs (with completeness $\geq$50% and contamination $\leq$5% as estimated by CheckM)[46] were classified at the level of genus using BAT.[47] This algorithm provides taxonomic affiliations to microbial genomes based on consensus taxa of proteins matches to the NCBI-nr database. Next, viral sequences were extracted from the SAGs using VIBRANT,[48] which identified 418 viral sequences. We assumed that the viral sequences in the SAGs infected the organisms from which these SAGs were derived, either because they were derived from integrated prophages or from viral particles attached or inside host cells. Viral sequences for which the host taxon predicted by RaFAH was the same taxon of the SAG as determined by BAT were considered as correct host predictions. Viruses from SAGs that could not be classified were excluded from the precision and recall analyses.

Test Set 3 comprised a collection of 61,647 viral genomic sequences from studies that spanned multiple samples from permafrost,[8] marine,[49] human gut,[50] freshwater,[19] soil,[51] hypersaline lakes,[52] hydrothermal springs (Fredrickson et al., unpublished data obtained from IMG/VR),[53] and sludge bioreactor[18] habitats. These sequences were assigned to putative hosts through the classical host-prediction pipeline described above for the GLUVAB genomes and also using RaFAH. Bootstrap analysis was applied to evaluate the precision of RaFAH in this dataset. For this, we assumed that the hosts predicted by the classical approaches were the true hosts of the viral genomes on Test Set 3. Random subsamples representing 20% of the full data were generated in 1,000 replicates. Precision was estimated for each replicate. Also, we estimated the completeness of viral genomes on Test Set 3 with CheckV[15] and analyzed the association between genome completeness and the precision of RaFAH.

RaFAH was tested on an Intel Xeon Gold 6140 CPU @ 2.30-GHz machine. Timing calculations were performed using randomly selected genomes of Test Set 3 using 24 threads in both the training and prediction modes (Figure S9). These results showed that the time to perform computations varied exponentially as a function of input genomes. Using 10,000 input genomes, RaFAH took 184 min to fit models and 495 min to predict hosts.

Comparison with other methods for host prediction

To assess the performance of RaFAH compared with other host-prediction tools, we assessed the performance of the alignment-free methods HostPhinder[5] and WIsH,[3] the alignment-dependent approaches based on homology matches, shared tRNAs and CRISPR spacers (and the three combined as described above for assigning hosts to GLUVAB genomes), and a hybrid approach, VirHostMatcher-Net.[6] We compared these tools on Test Sets 1, 2, and 3. HostPhinder, VirHostMatcher-Net, and WIsH were run with default parameters. The classical host predictions (CRISPR, tRNA, and homology matches) for Test Set 1 were performed using the same parameters described above for the GLUVAB genomes and for Test Set 3. Three performance metrics were evaluated at different taxonomic levels (domain to genus): Recall is the percentage of viral sequences for which a host was predicted by a given tool. Each viral sequence that was associated to a host was counted toward recall, regardless of the host association being correct or not. Recall was calculated as the number of sequences associated with a host divided by the total number of sequences in the dataset. For approaches that provided multiple host predictions for the same viral sequence (i.e., homology matches, tRNA, and CRISPR), each individual viral sequence counted toward recall only once. Precision is the percentage of host predictions that were correct. Each viral sequence that was associated with a host by a given tool was counted toward precision if the host association matched the true host of the sequence. Precision was calculated as the total of matching host predictions divided by the total number of predictions. Approaches that provided multiple host predictions for the same viral sequence counted toward precision if at least one

of the predictions was correct, but each sequence was counted toward precision only once. Finally, the F1 score was calculated as the harmonic mean between precision and recall.

For the approaches that required reference host genomes (i.e., WIsH, CRISPR, tRNA, and homology matches), the database of host genomes was the NCBI RefSeq genomes of Bacteria and Archaea and the genomes of Uncultured Bacteria and Archaea from the Genome Taxonomy Database.[54] To minimize false positives due to homology between viruses and mobile genetic elements, we removed all sequences that matched the keyword "plasmid" in their description field from the database of reference host genomes.

Assessment of archaeal virus-host predictions

To confirm the prediction of 537 genomes predicted by RaFAH as archaeal viruses, we used Mash v.2.1.[55] Mash calculates Jaccard distance between two genomes based on the number of shared *k*-mers with a certain length. We used *k*-mer sizes from 13 to 20 nucleotides. For each *k*-mer size we calculated distances of every phage genomic sequence against all potential host genomes. This database included 17,134 bacterial genomes and 4,716 archaeal genomes retrieved from RefSeq and GenBank. For each phage genome, we selected the potential host with the smallest Mash distance. In addition to Mash distance, we also calculated Manhattan distances and correlation scores between phage and host *k*-mer frequencies using $k = 6$ as described in Edwards et al.[2] and Ahlgren et al.[4] Finally, all 537 phages were used as BLASTn queries against the whole NR database. For each phage we determined a potential host by selecting the top-scoring non-viral hit as described in Edwards et al.[2] In addition, we compared the prevalence of ribosomal binding site motifs (defined by Prodigal[37] gene predictions) between viral sequences predicted to infect Bacteria and Archaea, from both the eight metagenomic datasets and RefSeq viruses. A similar analysis was performed to compare the prevalence of Pfam domains among these groups. For this analysis, protein sequences were queried against the Pfam database using hmmsearch with maximum e-value set to 10^{-3}.

SUPPLEMENTAL INFORMATION

Supplemental information can be found online at https://doi.org/10.1016/j.patter.2021.100274.

ACKNOWLEDGMENTS

This work was supported by grants "VIREVO" CGL2016-76273-P [MCI/AEI/FEDER, EU] (cofounded with FEDER funds) from the Spanish Ministerio de Ciencia e Innovación and "HIDRAS3" PROMETEU/2019/009 from Generalitat Valenciana. F.R.-V. was also a beneficiary of the 5top100-program of the Ministry for Science and Education of Russia. F.H.C. was supported by APOSTD/2018/186 post-doctoral fellowships from Generalitat Valenciana. A.Z. was funded by the Polish National Science Centre (2018/31/D/NZ2/00108). J.B.'s research was supported by the National Center for Research and Development (NCBR, Poland), grant number LIDER/5/0023/L-10/18/NCBR/2019. B.E.D. was supported by the Netherlands Organization for Scientific Research (NWO) Vidi grant 864.14.004 and by the European Research Council Consolidator grant 865694: DiversiPHI. R.E. was supported by National Institutes of Health grant RC2 DK116713-01A1.

AUTHOR CONTRIBUTIONS

F.H.C. conceived and designed the experiments. F.H.C., A.Z.-S., M.L.-P., A.Z., J.B., B.E.D., and R.E. analyzed the data. All authors contributed to writing the manuscript.

DECLARATION OF INTERESTS

The authors declare no competing interests.

Received: October 28, 2020
Revised: November 23, 2020
Accepted: May 7, 2021
Published: June 15, 2021

REFERENCES

1. Roux, S., Brum, J.R., Dutilh, B.E., Sunagawa, S., Duhaime, M.B., Loy, A., Poulos, B.T., Solonenko, N., Lara, E., Poulain, J., et al. (2016). Ecogenomics and biogeochemical impacts of uncultivated globally abundant ocean viruses. Nature *537*, 589–693.
2. Edwards, R.A., McNair, K., Faust, K., Raes, J., and Dutilh, B.E. (2016). Computational approaches to predict bacteriophage–host relationships. FEMS Microbiol. Rev. *40*, 258–272.
3. Galiez, C., Siebert, M., Enault, F., Vincent, J., and Söding, J. (2017). WIsH: who is the host? Predicting prokaryotic hosts from metagenomic phage contigs. Bioinformatics *33*, 3113–3114.
4. Ahlgren, N.A., Ren, J., Lu, Y.Y., Fuhrman, J.A., and Sun, F. (2017). Alignment-free d2* oligonucleotide frequency dissimilarity measure improves prediction of hosts from metagenomically-derived viral sequences. Nucleic Acids Res. *45*, 39–53.
5. Villarroel, J., Kleinheinz, K.A., Jurtz, V.I., Zschach, H., Lund, O., Nielsen, M., and Larsen, M.V. (2016). HostPhinder: a phage host prediction tool. Viruses *8*, 116.
6. Wang, W., Ren, J., Tang, K., Dart, E., Ignacio-Espinoza, J.C., Fuhrman, J.A., Braun, J., Sun, F., and Ahlgren, N.A. (2020). A network-based integrated framework for predicting virus–prokaryote interactions. NAR Genomics Bioinform. *2*, 505768.
7. Ignacio-Espinoza, J.C., Ahlgren, N.A., and Fuhrman, J.A. (2020). Long-term stability and Red Queen-like strain dynamics in marine viruses. Nat. Microbiol. *5*, 265–271.
8. Emerson, J.B., Roux, S., Brum, J.R., Bolduc, B., Woodcroft, B.J., Jang, H.B., Singleton, C.M., Solden, L.M., Naas, A.E., Boyd, J.A., et al. (2018). Host-linked soil viral ecology along a permafrost thaw gradient. Nat. Microbiol. *3*, 870–880.
9. López-Pérez, M., Haro-Moreno, J.M., Gonzalez-Serrano, R., Parras-Moltó, M., and Rodriguez-Valera, F. (2017). Genome diversity of marine phages recovered from Mediterranean metagenomes: size matters. PLoS Genet. *13*, e1007018.
10. Zhang, M., Yang, L., Ren, J., Ahlgren, N.A., Fuhrman, J.A., and Sun, F. (2017). Prediction of virus-host infectious association by supervised learning methods. BMC Bioinformatics *18*, 60.
11. Young, F., Rogers, S., and Robertson, D.L. (2020). Predicting host taxonomic information from viral genomes: a comparison of feature representations. PLoS Comput. Biol. *16*, e1007894.
12. Leite, D.M.C., Brochet, X., Resch, G., Que, Y.-A., Neves, A., and Peña-Reyes, C. (2018). Computational prediction of inter-species relationships through omics data analysis and machine learning. BMC Bioinformatics *19*, 420.
13. de Jonge, P.A., Nobrega, F.L., Brouns, S.J.J., and Dutilh, B.E. (2018). Molecular and evolutionary determinants of bacteriophage host-range. Trends Microbiol. *27*, 51–63.
14. Pachiadaki, M.G., Brown, J.M., Brown, J., Bezuidt, O., Berube, P.M., Biller, S.J., Poulton, N.J., Burkart, M.D., La Clair, J.J., Chisholm, S.W., et al. (2019). Charting the complexity of the marine microbiome through single-cell genomics. Cell *179*, 1623–1635.e11.
15. Nayfach, S., Camargo, A.P., Schulz, F., Eloe-Fadrosh, E., Roux, S., and Kyrpides, N.C. (2020). CheckV assesses the quality and completeness of metagenome-assembled viral genomes. Nat. Biotechnol. https://doi.org/10.1038/s41587-020-00774-7.
16. Sunagawa, S., Coelho, L.P., Chaffron, S., Kultima, J.R., Labadie, K., Salazar, G., Djahanschiri, B., Zeller, G., Mende, D.R., Alberti, A., et al. (2015). Structure and function of the global ocean microbiome. Science *348*, 1261359.
17. Arumugam, M., Raes, J., Pelletier, E., Le Paslier, D., Yamada, T., Mende, D.R., Fernandes, G.R., Tap, J., Bruls, T., Batto, J.-M., et al. (2011). Enterotypes of the human gut microbiome. Nature *473*, 174–180.

CellPress
OPEN ACCESS

18. Liu, R., Qi, R., Wang, J., Zhang, Y., Liu, X., Rossetti, S., Tandoi, V., and Yang, M. (2017). Phage-host associations in a full-scale activated sludge plant during sludge bulking. Appl. Microbiol. Biotechnol. *101*, 6495–6504.
19. Kavagutti, V.S., Andrei, A.-Ş., Mehrshad, M., Salcher, M.M., and Ghai, R. (2019). Phage-centric ecological interactions in aquatic ecosystems revealed through ultra-deep metagenomics. Microbiome *7*, 135.
20. Johnston, E.R., Hatt, J.K., He, Z., Wu, L., Guo, X., Luo, Y., Schuur, E.A.G., Tiedje, J.M., Zhou, J., and Konstantinidis, K.T. (2019). Responses of tundra soil microbial communities to half a decade of experimental warming at two critical depths. Proc. Natl. Acad. Sci. *116*, 201901307.
21. Ghai, R., Pašić, L., Fernández, A.B., Martin-Cuadrado, A.B., Mizuno, C.M., McMahon, K.D., Papke, R.T., Stepanauskas, R., Rodriguez-Brito, B., Rohwer, F., et al. (2011). New abundant microbial groups in aquatic hypersaline environments. Sci. Rep. *1*, 135.
22. Menzel, P., Gudbergsdóttir, S.R., Rike, A.G., Lin, L., Zhang, Q., Contursi, P., Moracci, M., Kristjansson, J.K., Bolduc, B., Gavrilov, S., et al. (2015). Comparative metagenomics of eight geographically remote terrestrial hot springs. Microb. Ecol. *70*, 411–424.
23. Philosof, A., Yutin, N., Flores-Uribe, J., Sharon, I., Koonin, E.V., and Béjà, O. (2017). Novel abundant oceanic viruses of uncultured marine group II Euryarchaeota identified by genome-centric metagenomics. Curr. Biol. *27*, 1362–1368.
24. Vik, D.R., Roux, S., Brum, J.R., Bolduc, B., Emerson, J.B., Padilla, C.C., Stewart, F.J., and Sullivan, M.B. (2017). Putative archaeal viruses from the mesopelagic ocean. PeerJ *5*, e3428.
25. Ahlgren, N.A., Fuchsman, C.A., Rocap, G., and Fuhrman, J.A. (2019). Discovery of several novel, widespread, and ecologically distinct marine Thaumarchaeota viruses that encode amoC nitrification genes. ISME J. *13*, 618–631.
26. López-Pérez, M., Haro-Moreno, J.M., de la Torre, J.R., and Rodriguez-Valera, F. (2019). Novel caudovirales associated with marine group I Thaumarchaeota assembled from metagenomes. Environ. Microbiol. *21*, 1980–1988.
27. Marine, R.L., Nasko, D.J., Wray, J., Polson, S.W., and Wommack, K.E. (2017). Novel chaperonins are prevalent in the virioplankton and demonstrate links to viral biology and ecology. ISME J. *11*, 2479–2491.
28. Turner, B., Burkhart, B.W., Weidenbach, K., Ross, R., Limbach, P.A., Schmitz, R.A., de Crécy-Lagard, V., Stedman, K.M., Santangelo, T.J., and Iwata-Reuyl, D. (2020). Archaeosine modification of archaeal tRNA: role in structural stabilization. J. Bacteriol. *202*. https://doi.org/10.1128/JB.00748-19.
29. Makarova, K.S., and Koonin, E.V. (2010). Archaeal ubiquitin-like proteins: functional versatility and putative ancestral involvement in tRNA modification revealed by comparative genomic analysis. Archaea *2010*, 9–13.
30. Randow, F., and Lehner, P.J. (2009). Viral avoidance and exploitation of the ubiquitin system. Nat. Cell Biol. *11*, 527–534.
31. Fu, L., Niu, B., Zhu, Z., Wu, S., and Li, W. (2012). CD-HIT: accelerated for clustering the next-generation sequencing data. Bioinformatics *28*, 3150–3152.
32. Coutinho, F.H., Edwards, R.A., and Rodríguez-Valera, F. (2019). Charting the diversity of uncultured viruses of Archaea and Bacteria. BMC Biol. *17*, 109.
33. Díez-Villaseñor, C., and Rodriguez-Valera, F. (2019). CRISPR analysis suggests that small circular single-stranded DNA smacoviruses infect Archaea instead of humans. Nat. Commun. *10*, 294.
34. Altschul, S.F., Gish, W., Miller, W., Myers, E.W., and Lipman, D.J. (1990). Basic local alignment search tool. J. Mol. Biol. *215*, 403–410.
35. Lowe, T.M., and Chan, P.P. (2016). tRNAscan-SE On-line: integrating search and context for analysis of transfer RNA genes. Nucleic Acids Res. *44*, W54–W57.
36. Brum, J.R., Ignacio-Espinoza, J.C., Roux, S., Doulcier, G., Acinas, S.G., Alberti, A., Chaffron, S., Cruaud, C., de Vargas, C., Gasol, J.M., et al. (2015). Patterns and ecological drivers of ocean viral communities. Science *348*, 1261498.
37. Hyatt, D., Chen, G.-L., Locascio, P.F., Land, M.L., Larimer, F.W., and Hauser, L.J. (2010). Prodigal: prokaryotic gene recognition and translation initiation site identification. BMC Bioinformatics *11*, 119.
38. Steinegger, M., and Söding, J. (2017). MMseqs2 enables sensitive protein sequence searching for the analysis of massive data sets. Nat. Biotechnol. *35*, 1026–1028.
39. Gudyś, A., and Deorowicz, S. (2017). QuickProbs 2: towards rapid construction of high-quality alignments of large protein families. Sci. Rep. *7*, 41553.
40. Finn, R.D., Clements, J., Arndt, W., Miller, B.L., Wheeler, T.J., Schreiber, F., Bateman, A., and Eddy, S.R. (2015). HMMER web server: 2015 update. Nucleic Acids Res. *43*, W30–W38.
41. Grazziotin, A.L., Koonin, E.V., and Kristensen, D.M. (2017). Prokaryotic Virus Orthologous Groups (pVOGs): a resource for comparative genomics and protein family annotation. Nucleic Acids Res. *45*, D491–D498.
42. Steinegger, M., Meier, M., Mirdita, M., Vöhringer, H., Haunsberger, S.J., and Söding, J. (2019). HH-suite3 for fast remote homology detection and deep protein annotation. BMC Bioinformatics *20*, 473.
43. Wright, M.N., and Ziegler, A. (2017). ranger: A fast implementation of random forests for high dimensional data in C++ and R. J. Stat. Softw. *77*. https://doi.org/10.18637/jss.v077.i01.
44. R Core Team (2016). R : A Language and Environment for Statistical Computing (R Foundation for Statistical Computing).
45. Buchfink, B., Xie, C., and Huson, D.H. (2015). Fast and sensitive protein alignment using DIAMOND. Nat. Methods *12*, 59–60.
46. Parks, D.H., Imelfort, M., Skennerton, C.T., Hugenholtz, P., and Tyson, G.W. (2015). CheckM: assessing the quality of microbial genomes recovered from isolates, single cells, and metagenomes. Genome Res. *25*, 1043–1055.
47. von Meijenfeldt, F.A.B., Arkhipova, K., Cambuy, D.D., Coutinho, F.H., and Dutilh, B.E. (2019). Robust taxonomic classification of uncharted microbial sequences and bins with CAT and BAT. Genome Biol. *20*, 530188.
48. Kieft, K., Zhou, Z., and Anantharaman, K. (2020). VIBRANT: automated recovery, annotation and curation of microbial viruses, and evaluation of viral community function from genomic sequences. Microbiome *8*, 90.
49. Luo, E., Eppley, J.M., Romano, A.E., Mende, D.R., and DeLong, E.F. (2020). Double-stranded DNA virioplankton dynamics and reproductive strategies in the oligotrophic open ocean water column. ISME J. *14*, 1304–1315.
50. Gregory, A.C., Zablocki, O., Howell, A., Bolduc, B., and Sullivan, M.B. (2019). The human gut virome database. bioRxiv. https://doi.org/10.1101/655910.
51. Göller, P.C., Haro-Moreno, J.M., Rodriguez-Valera, F., Loessner, M.J., and Gómez-Sanz, E. (2020). Uncovering a hidden diversity: optimized protocols for the extraction of dsDNA bacteriophages from soil. Microbiome *8*, 17.
52. Roux, S., Enault, F., Ravet, V., Colombet, J., Bettarel, Y., Auguet, J.C., Bouvier, T., Lucas-Staat, S., Vellet, A., Prangishvili, D., et al. (2016). Analysis of metagenomic data reveals common features of halophilic viral communities across continents. Environ. Microbiol. *18*, 889–903.
53. Paez-Espino, D., Roux, S., Chen, I.-M.A., Palaniappan, K., Ratner, A., Chu, K., Huntemann, M., Reddy, T.B.K., Pons, J.C., Llabrés, M., et al. (2018). IMG/VR v.2.0: an integrated data management and analysis system for cultivated and environmental viral genomes. Nucleic Acids Res. *47*, 678–686.
54. Parks, D.H., Chuvochina, M., Waite, D.W., Rinke, C., Skarshewski, A., Chaumeil, P.-A., and Hugenholtz, P. (2018). A standardized bacterial taxonomy based on genome phylogeny substantially revises the tree of life. Nat. Biotechnol. *36*, 996–1004.
55. Ondov, B.D., Treangen, T.J., Melsted, P., Mallonee, A.B., Bergman, N.H., Koren, S., and Phillippy, A.M. (2016). Mash: fast genome and metagenome distance estimation using MinHash. Genome Biol. *17*, 132.

Patterns, Volume *2*

Supplemental information

RaFAH: Host prediction for viruses of Bacteria and Archaea based on protein content

Felipe Hernandes Coutinho, Asier Zaragoza-Solas, Mario López-Pérez, Jakub Barylski, Andrzej Zielezinski, Bas E. Dutilh, Robert Edwards, and Francisco Rodriguez-Valera

Supplementary material:

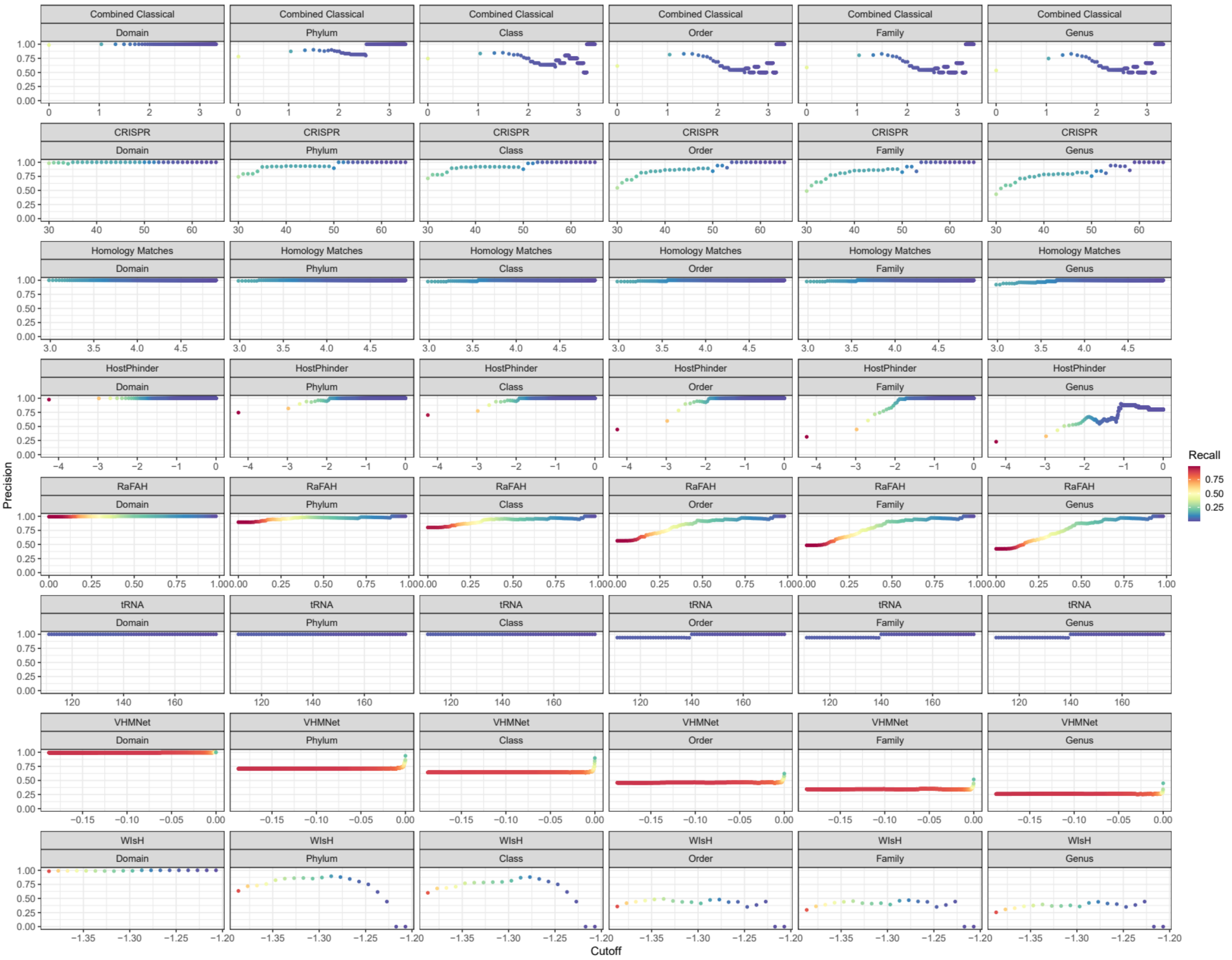

Figure S1: Associations between score cutoff, precision, and recall for RaFAH, the alignment-free (WIsH and HostPhinder), hybrid (VirHostMatcher-Net), and classical (CRISPR, tRNA and homology matches) host prediction approaches on Test Set 1. The score cutoffs for HostPhinder, Homology matches, VirHostMatcher-Net and Combined Classical are shown in the Log10 scale.

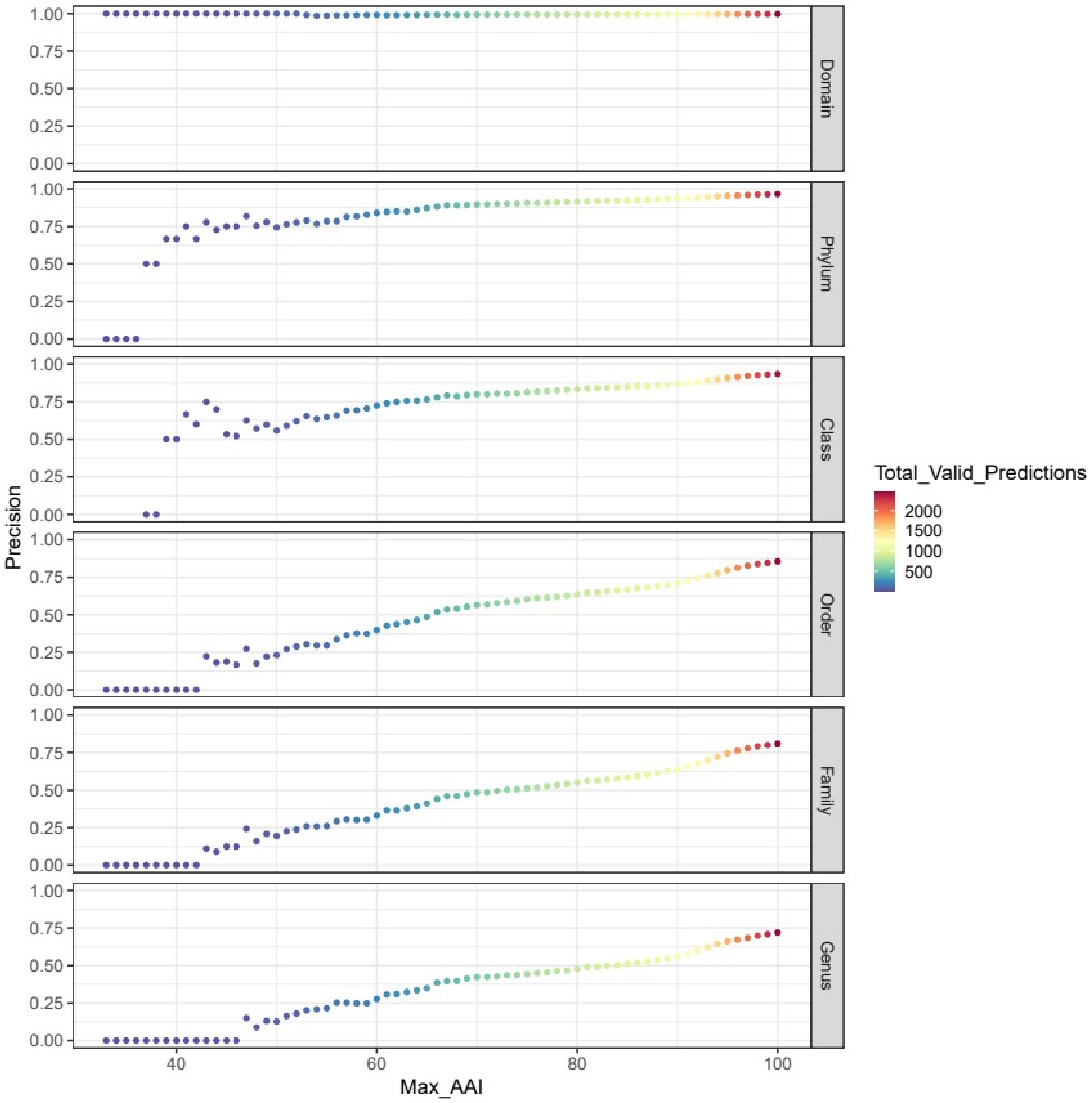

Figure S2: Associations between precision and similarity among Test Set 1 and Training Set 3 genomes. Each panel represents a different taxonomic level. X axis displays the maximum Average Amino acid Identity (AAI) among genomes of the two sets. Y axis displays the precision of RaFAH. Points are coloured according to the number of valid predictions (host taxon predicted by RaFAH for a non “NA/undef/Unknown” host genome in Test Set 1) yielded at each taxonomic level and AAI cutoff. For this particular analysis all non-redundant genomes in Test Set 1 were used while in all other instances this dataset was filtered for maximum 70% AAI and 70% matched proteins.

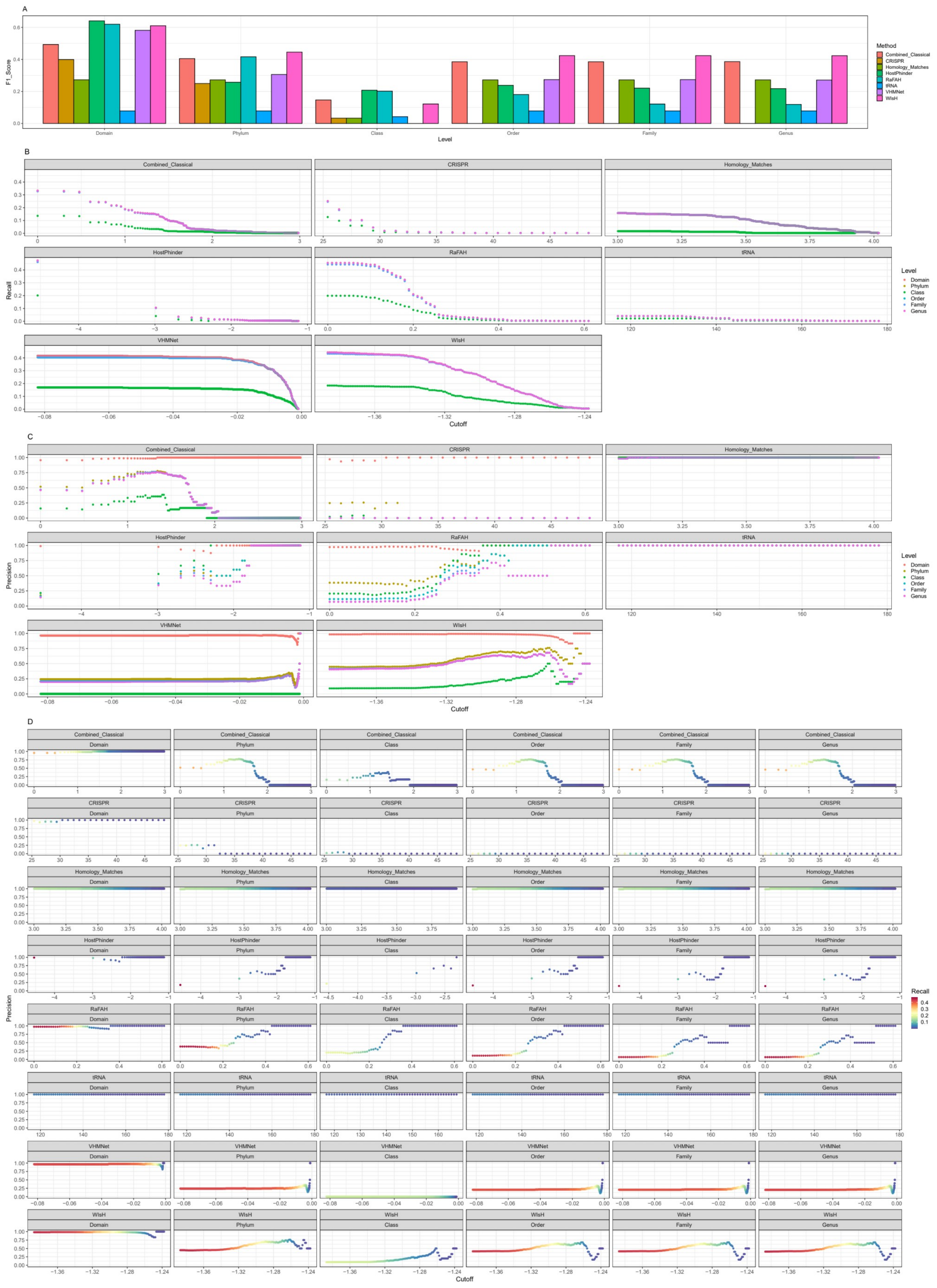

Figure S3: Performance of host prediction tools on Test Set 2: Associations between score cutoff, precision, and recall for RaFAH, the alignment-free (WIsH and HostPhinder), hybrid

(VirHostMatcher-Net), and classical (CRISPR, tRNA and homology matches) host prediction approaches. A) F1-score of methods when considering all predictions regardless of score at multiple taxonomic levels. B) Association between score cutoff and recall for each taxonomic level. C) Association between score cutoff and precision for each taxonomic level D) Associations between precision and recall in function of score cutoff. Figure S8 depicts the association between precision and score cutoff of VirHostMatcher-Net for score values above the 75th percentile.

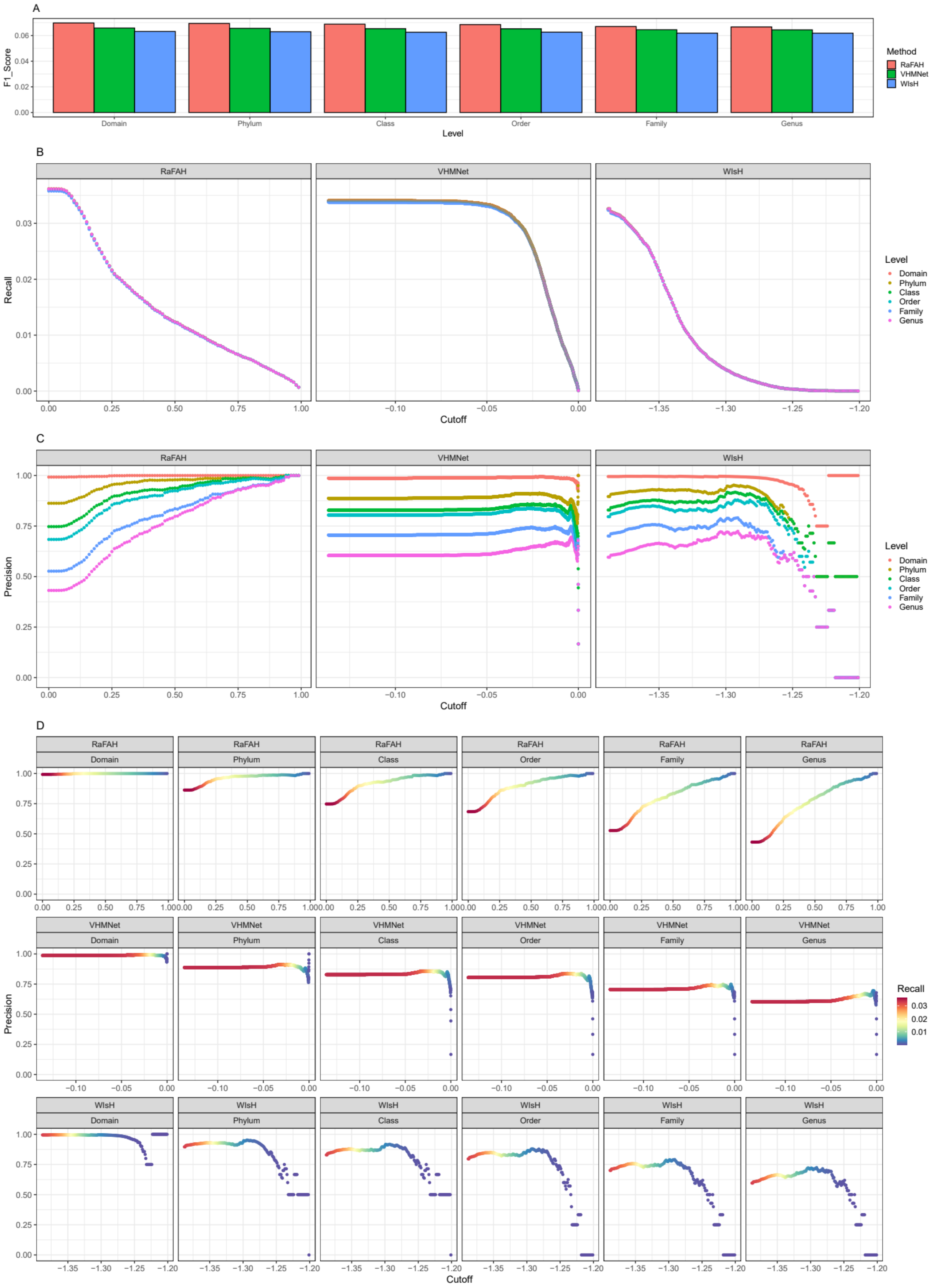

Figure S4: Performance of host prediction tools on Test Set 3: Associations between score cutoff, precision, and recall for RaFAH, WIsH and VirHostMatcher-Net. The hosts assigned by the

combined classical approaches were considered the true hosts of the genomes in Test Set 3. A) F1-score of methods when considering all predictions regardless of score at multiple taxonomic levels. B) Association between score cutoff and recall for each taxonomic level. C) Association between score cutoff and precision for each taxonomic level. D) Associations between precision and recall in function of score cutoff. Figure S8 depicts the association between precision and score cutoff of VirHostMatcher-Net for score values above the 75th percentile.

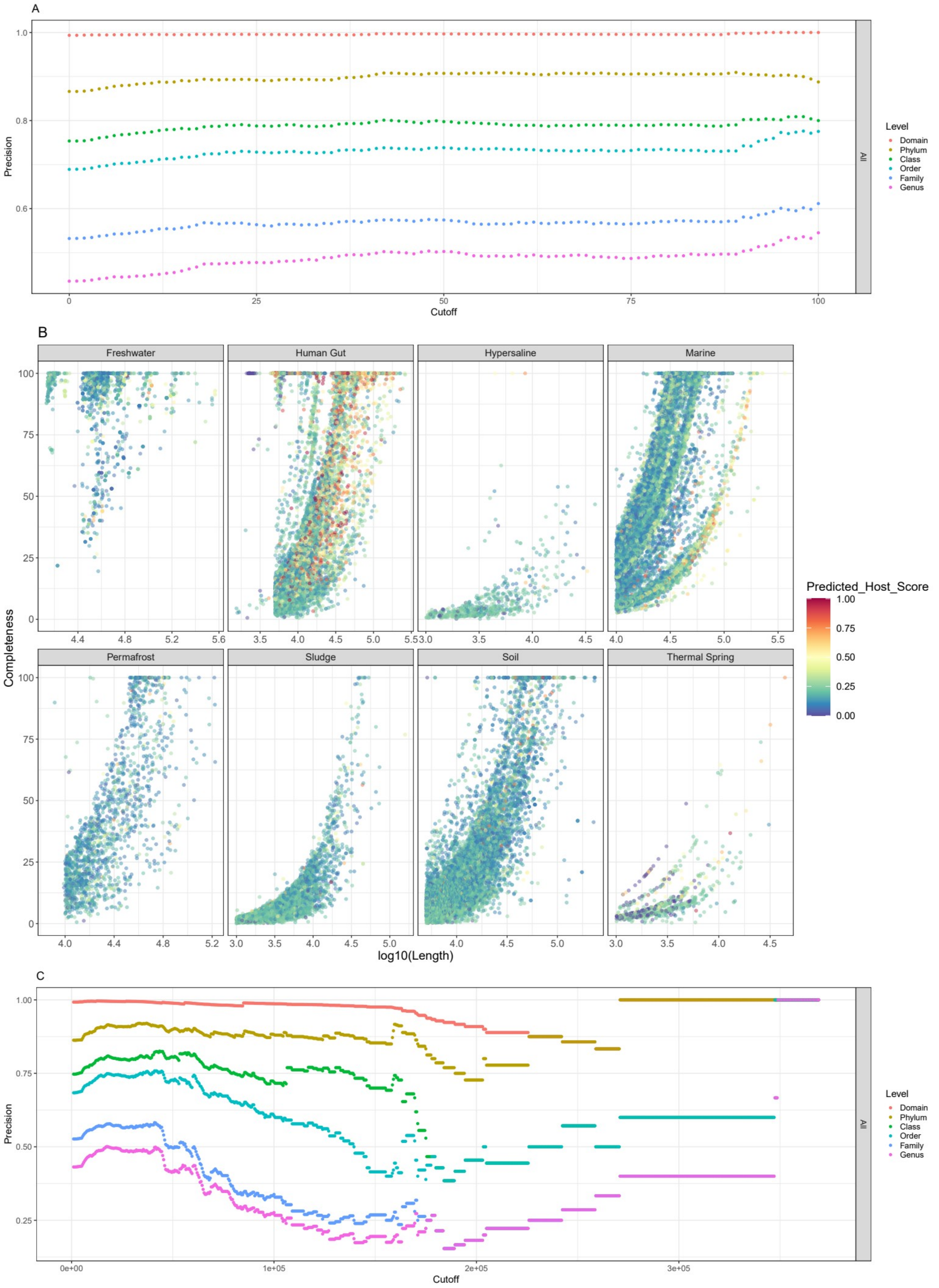

Figure S5: Associations between the performance of RaFAH and genome length/completeness on Test Set 3 genomes. A) Scatterplot displaying the cutoff for genome completeness (X axis) and

precision of RaFAH (y axis). B) Association between genome legngth (X axis) and genome completeness (Y axis) estimated with CheckV across 8 ecosystems (Panels). C) Scatterplot displaying the cutoff for genome length (X axis) and precision of RaFAH (y axis).

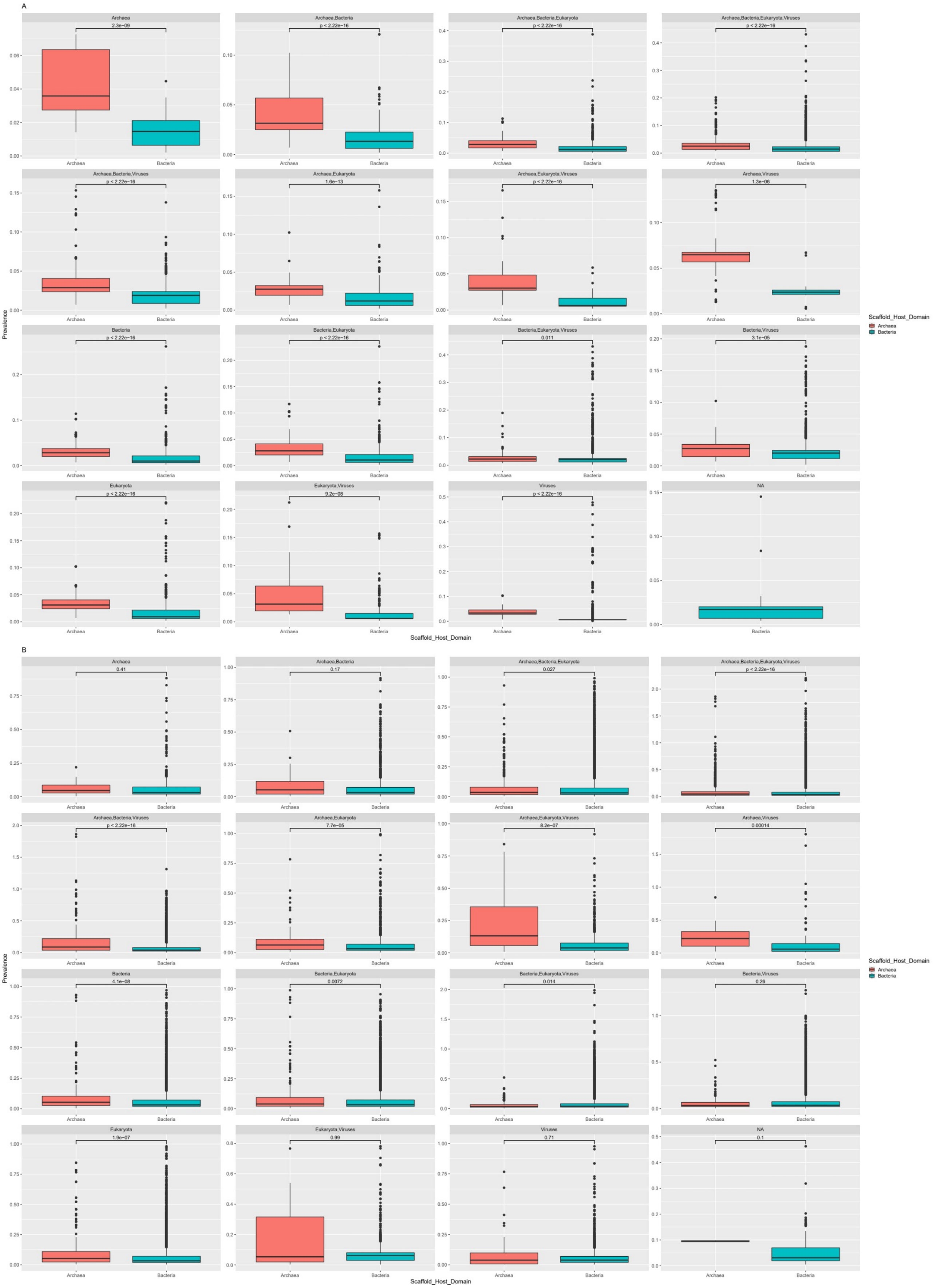

Figure S6: Prevalence of Pfam domains among viruses. Pfam domains were grouped according to their expected taxonomic ranges (depicted above each panel). Only values derived from scaffolds

with at least 5 CDS are shown to reduce noise. A) Comparisons of Pfam domain prevalence between RefSeq viruses of Archaea and Bacteria. The *p* values of each comparison obtained with the Mann-Whitney test are depicted above bars. B) Pfam domain prevalence between RefSeq viruses of Archaea and of Bacteria from TestSet3. Notice the different y axes on each panel.

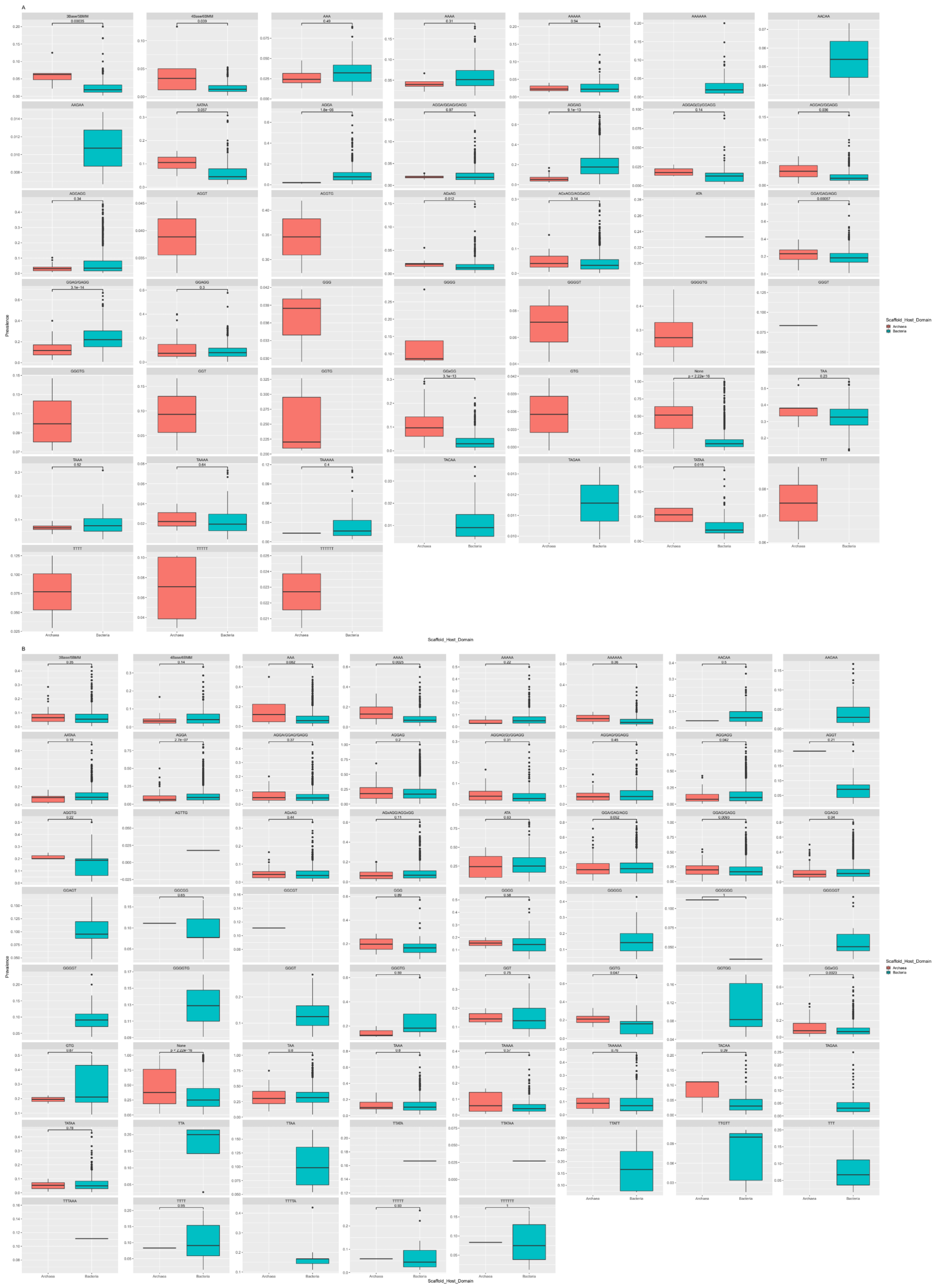

Figure S7: Prevalence of ribosomal binding site (RBS) motifs among viruses. Only values derived from scaffolds with at least 5 CDS are shown to reduce noise. A) Comparisons of RBS motif

prevalence between RefSeq viruses of Archaea and Bacteria. The *p*-values of each comparison obtained with the Mann-Whitney test are depicted above bars. B) Comparisons RBS motif prevalence between RefSeq viruses of Archaea and Bacteria and viruses from TestSet3.

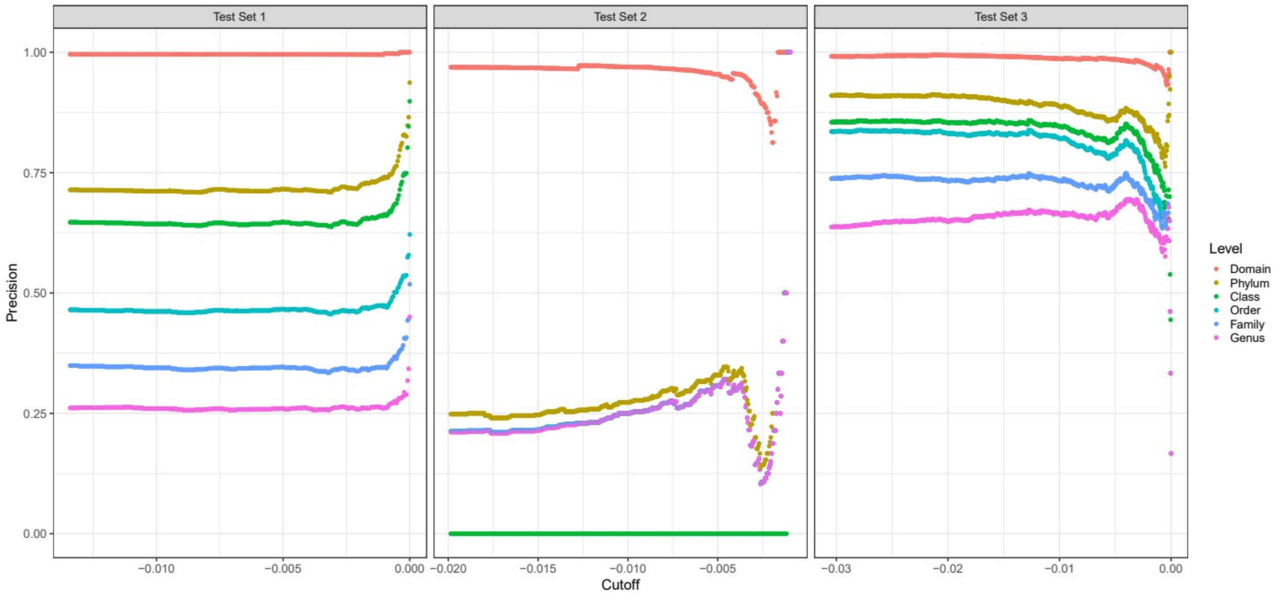

Figure S8: Association between precision and score cutoff for VirHostMatcher-Net in Test Sets 1, 2 and 3. All scores below the 75th percentile value were excluded from this analysis.

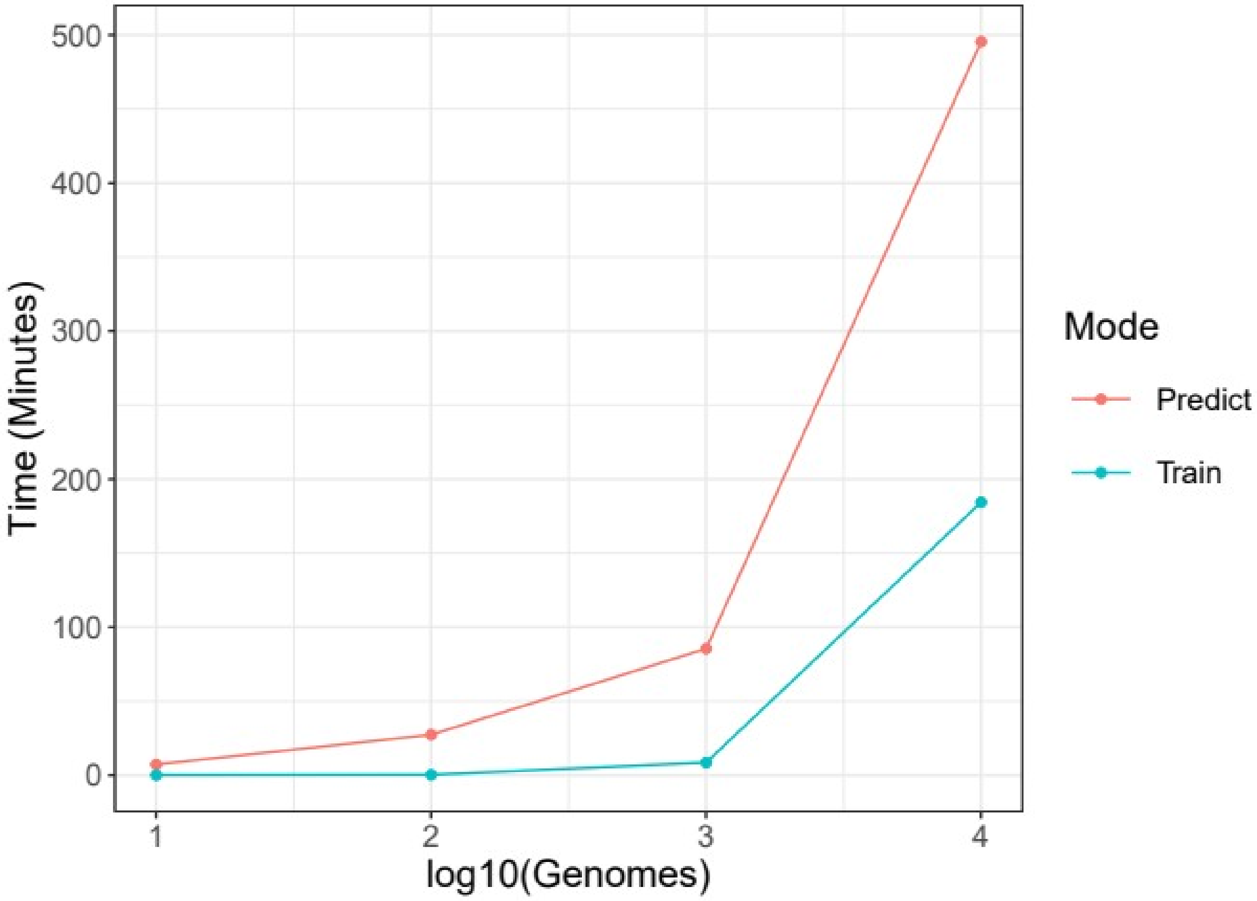

Figure S9: Timing of RaFAH computation on Training and Prediction modes (Y axis) as a function of the number of input genomes (X axis). Calculations were performed using randomly selected genomes of Test Set 3 on an Intel(R) Xeon(R) Gold 6140 CPU @ 2.30GHz machine using 24 threads.

Supplementary Data 1: Fasta file containing the nucleotide sequences from NCBI RefSeq and GLUVAB viruses that made up Training Sets 1, 2, and 3. Due to file size Supplementary Data 1 is available on Figshare with DOI: https://doi.org/10.6084/m9.figshare.14208500

Supplementary Data 2: Fasta file containing the nucleotide sequences from NCBI RefSeq that made up Testing Set 1. Due to file size Supplementary Data 2 is available on Figshare with DOI: https://doi.org/10.6084/m9.figshare.14210591

Supplementary Data 3: Fasta file containing the nucleotide sequences from SAG derived viruses that made up Testing Set 2. Due to file size Supplementary Data 3 is available on Figshare with DOI: https://doi.org/10.6084/m9.figshare.14208506

Supplementary Data 4: Fasta file containing the nucleotide sequences from metagenome derived viruses that made up Testing Set 3. Due to file size Supplementary Data 4 is available on Figshare with DOI: https://doi.org/10.6084/m9.figshare.14210612
